# Modulation of STAT3 Signaling, Cell Redox Defenses and Cell Cycle Checkpoints by β-Caryophyllene in Cholangiocarcinoma Cells: Possible Mechanisms Accounting for Doxorubicin Chemosensitization and Chemoprevention

**DOI:** 10.3390/cells9040858

**Published:** 2020-04-02

**Authors:** Antonella Di Sotto, Silvia Di Giacomo, Elisabetta Rubini, Alberto Macone, Marco Gulli, Caterina Loredana Mammola, Margherita Eufemi, Romina Mancinelli, Gabriela Mazzanti

**Affiliations:** 1 Department of Physiology and Pharmacology “V. Erspamer”, Sapienza University of Rome, P.le Aldo Moro 5, 00185 Rome, Italy; silvia.digiacomo@uniroma1.it (S.D.G.); marco.gulli@uniroma1.it (M.G.); gabriela.mazzanti@uniroma1.it (G.M.); 2 Department of Biochemical Science “A. Rossi Fanelli”, Sapienza University of Rome, P.le Aldo Moro 5, 00185 Rome, Italy; elisabetta.rubini@uniroma1.it (E.R.); alberto.macone@uniroma1.it (A.M.); margherita.eufemi@uniroma1.it (M.E.); 3 Department of Anatomical, Histological, Forensic and Orthopedic Sciences, Sapienza University of Rome, P.le Aldo Moro 5, 00185 Rome, Italy; caterinaloredana.mammola@uniroma1.it (C.L.M.); romina.mancinelli@uniroma1.it (R.M.)

**Keywords:** chemoprevention, genoprotective effects, caryophyllane sesquiterpenes, liver cancer, metronomic schedule, GSH depletion, apoptosis, H2AX phosphorylation, cholangiocytes, cell cycle checkpoint, STAT3 signaling

## Abstract

Cholangiocarcinoma (CCA) is an aggressive group of biliary tract cancers, characterized by late diagnosis, low effective chemotherapies, multidrug resistance, and poor outcomes. In the attempt to identify new therapeutic strategies for CCA, we studied the antiproliferative activity of a combination between doxorubicin and the natural sesquiterpene β-caryophyllene in cholangiocarcinoma Mz-ChA-1 cells and nonmalignant H69 cholangiocytes, under both long-term and metronomic schedules. The modulation of STAT3 signaling, oxidative stress, DNA damage response, cell cycle progression and apoptosis was investigated as possible mechanisms of action. β-caryophyllene was able to synergize the cytotoxicity of low dose doxorubicin in Mz-ChA-1 cells, while producing cytoprotective effects in H69 cholangiocytes, mainly after a long-term exposure of 24 h. The mechanistic analysis highlighted that the sesquiterpene induced a cell cycle arrest in G2/M phase along with the doxorubicin-induced accumulation in S phase, reduced the γH2AX and GSH levels without affecting GSSG. ROS amount was partly lowered by the combination in Mz-ChA-1 cells, while increased in H69 cells. A lowered expression of doxorubicin-induced STAT3 activation was found in the presence of β-caryophyllene in both cancer and normal cholangiocytes. These networking effects resulted in an increased apoptosis rate in Mz-ChA-1 cells, despite a lowering in H69 cholangiocytes. This evidence highlighted a possible role of STAT3 as a final effector of a complex network regulated by β-caryophyllene, which leads to an enhanced doxorubicin-sensitivity of cholangiocarcinoma cells and a lowered chemotherapy toxicity in nonmalignant cholangiocytes, thus strengthening the interest for this natural sesquiterpene as a dual-acting chemosensitizing and chemopreventive agent.

## 1. Introduction

Cholangiocarcinoma (CCA) represents a large group of epithelial cancers originated from cholangiocytes, the epithelial cells lining the biliary tree and classified as intrahepatic, perihilar and distal types based on their anatomic location [1,2]. Owing to its asymptomatic growth, CCA is often diagnosed at advanced stages, when chemotherapy remains the unique curative approach, although with a lower than 10% survival rate [3]. At the moment, gemcitabine plus cisplatin is the reference chemotherapy regimen for CCA, but other drugs, such as doxorubicin, sorafenib and regorafenib, are commonly used, although with low success due to the severe toxicity and multidrug resistance (MDR) development [4,5,6]. The intrinsic resistance of normal cholangiocytes to drug cytotoxicity is considered a key feature of CCA cells responsible for the chemotherapy resistance [7]. This evidence highlights the imperative need to develop more sophisticated therapeutic tools to improve survival rates of CCA patients, by increasing chemotherapy efficacy while limiting side effects and chemoresistance development.

Among alternative chemotherapies, doxorubicin is considered a valuable strategy for liver cancer, especially when administered as advanced pharmaceutical forms or in polytherapy regimens [8], because of its powerful cancer-killing activity, based on multiple cytotoxic mechanisms (i.e., DNA-damage, block of cancer cell cycle progression and increase of intracellular oxidative stress) [9,10]. Nevertheless, its low oral bioavailability requires the drug is administered by a single intravenous injection of high doses, leading to severe side effects on normal tissues, that often hinder the continuation of chemotherapy [11,12]. In the attempt to overcome these drawbacks, alternative strategies for doxorubicin administration have been approached, achieving a similar efficacy and increased tolerability [13,14,15]. Recently, alternative pharmacological regimens, such as a metronomic chemotherapy (i.e., drug administration at low doses and more frequent intervals) and a combination therapy (co-administration of different drugs with various mechanisms and targets) have been proposed as innovative strategies to retain chemotherapy efficacy but limiting the occurrence of side effects and complications [16,17].

Combining an anticancer drug with a chemosensitizer has been found able to induce synergistic or additive interactions and to reduce the likelihood of drug resistance and systemic toxicity [18]. Drug combination is already applied successfully in the treatment of cancer and other diseases [19] and has been suggested to be a suitable strategy for poor prognosis cancers, including liver cancer [17]. A number of natural substances have been found able to synergize *in vitro* chemotherapeutic drugs and to resensitize resistant tumor cells by reversing MDR (e.g., curcumin, flavonoids) [20,21,22,23]. Our previous studies have highlighted a potential interest for the natural caryophyllane sesquiterpenes as chemosensitizing agents in different cancer cell lines [24,25,26].

Caryophyllane sesquiterpenes are natural phytochemicals characterized by a unique bicyclic structure with a rare dimethylcyclobutane ring fused in a trans configuration to a nine-carbon ring containing a 1,5-diene [27]. They are known to possess a safe toxicity profile [28,29,30] and to be devoid of genotoxic effects [30,31,32,33]. Particularly, β-caryophyllene is widely approved as a food additive and as a cosmetic ingredient [28], due to its very low toxicity as shown in *in vivo* studies [34,35,36]. β-Caryophyllene also exhibited pleiotropic pharmacological activities in preclinical studies [37]. It acts as an agonist of cannabinoid CB2 and PPAR (peroxisome proliferator activated receptor) receptors, thus leading to beneficial effects on several diseases, such as neuroinflammation, neurodegenerative pathologies and some types of cancer [37]. Furthermore, it produces cytoprotective effects by modulating oxidative stress, apoptosis and inflammation [38,39,40,41], through the interference with different inflammatory pathways, such as the inducible nitric oxide synthase (iNOS), tumor necrosis factor-alfa (TNF-α) and nuclear factor-κB (NF-κB) [37]. Also, it exhibited chemopreventive properties, such as genoprotective and antiproliferative ones, by inhibiting DNA damage and STAT3 (signal transducer and activator of transcription 3) activation induced by environmental pollutants [31,32,42,43] and through affecting multiple cascades involved in cancer growth [37,44,45,46,47]. Similar properties have been also reported for the metabolite β-caryophyllene oxide [48,49]. Recently, we demonstrated that caryophyllane sesquiterpenes are able to synergistically potentiate the antiproliferative effects of doxorubicin in human hepatoblastoma HepG2 cells both in standard long-term and metronomic treatments [26]: this suggests that combining the chemosensitization by caryophyllane sesquiterpenes and a metronomic schedule can be a smart strategy to overcome the drawbacks of doxorubicin chemotherapy while exploiting its powerful activity to conquer liver cancer [26].

**Figure 1:**
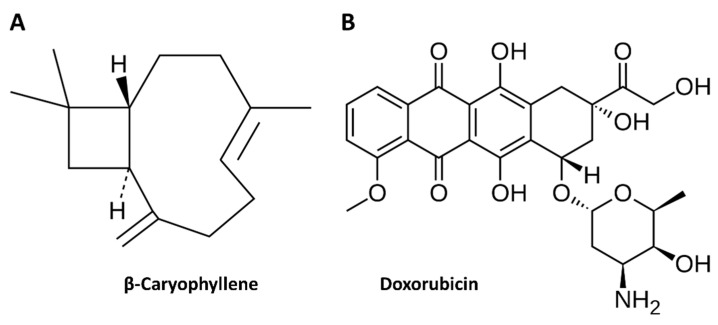
Molecular structure of the natural sesquiterpene β-caryophyllene (**A**) and the anticancer drug doxorubicin (**B**).

In line with previous evidence about the chemosensitizing properties of caryophyllane sesquiterpenes [24,25,26,50], in the present study we evaluated the ability of β-caryophyllene to synergize doxorubicin (Figure 1) in Mz-ChA-1 cholangiocarcinoma cells under both long-term and metronomic exposure schedules. Furthermore, being β-caryophyllene known to be protective in normal tissue against several toxicants [31,32,38,39,40,41,42,43], its ability to reduce doxorubicin toxicity in H69 noncancerous cholangiocytes, under the same exposure schedules applied for the doxorubicin chemosensitization, was assessed too. This could represent an important goal to overcome the toxicity drawback of doxorubicin chemotherapy while maintaining its anticancer efficacy.

In order to characterize the possible mechanisms accounting for the chemopreventive and chemosensitizing effects of β-caryophyllene towards doxorubicin in normal and cancer cells, different cellular parameters, including genotoxic damage, cell cycle progression, intracellular oxidative stress and apoptosis extent, that mediate doxorubicin cytotoxicity [10], were measured. Particularly, the level of genotoxic damage was determined in term of phosphorylation of histone 2AX at the serine 139 (Ser139) residue, namely γH2AX, which is known to occur in response to DNA double-strand break [51], thus being a suitable marker of DNA damage. Oxidative stress was characterized by measuring the increased intracellular ROS levels and glutathione defenses. Furthermore, being the dysregulation of cell division a hallmark of cancer cell growth and survival [52], the ability of the test substances to target the cell cycle machinery was measured too. In support, modulators of cell cycle checkpoints, alone or in combination with standard anticancer drugs, have been proposed as possible new strategies against cancer [53].

Oxidative stress, DNA damage and apoptosis are closely regulated by STAT3 [54], a cytoplasmic transcription factors directly implicated in CCA carcinogenesis and also considered as a marker of CCA poor prognosis [55,56,57]. A feedback activation of STAT3 has been also found engaged by anticancer drugs, like doxorubicin, leading to drug-resistance [58]. In this context, we also evaluated if a modulation of STAT3 activation could mediate the chemosensitizing and chemopreventive effects of β-caryophyllene in normal and cancer cholangiocytes.

## 2. Materials and Methods

### 2.1. Chemical and Reagents

All the substances, if not otherwise specified, were purchased from Sigma Aldrich Co (St. Louis, MO, USA). β-caryophyllene (Figure 1A), doxorubicin hydrochloride (Figure 1B) and ethanol (EtOH) were ≥ 98.0% purity. CMRL 1066 medium and Dulbecco’s Modified Eagle’s Medium Ham’s F12 (DMEM- F12) were provided by Aurogene (Rome, Italy). The sources of antibodies and materials for molecular biology analysis were specified in the relative paragraphs. To perform the experiments, all the solutions were prepared in the suitable solvent and sterilized by filtration using 0.2 µm pore-size cellulose acetate filters. β-caryophyllene and doxorubicin were dissolved in EtOH 100% *v*/*v* and deionized water, respectively, hence diluted in the complete medium. EtOH was used at a maximum 1% *v*/*v* nontoxic concentration in the medium.

### 2.2. Cell Cultures

An extrahepatic cholangiocarcinoma Mz-ChA-1 cell line, along with the nonmalignant H69 cholangiocytes, were used. Mz-ChA-1 cells from human gallbladder were a gift of Prof. G. Alpini (Indiana University School of Medicine, Indianapolis, IN). The cells were maintained at 37 °C in a 5% CO_2_ incubator in a culture medium composed of CMRL 1066 medium supplemented with 10% fetal bovine serum (FBS), 1% penicillin, gentamycin and streptomycin and 2 mM glutamine. The typical medium for the nonmalignant cholangiocytes (another gift of Prof. G. Alpini) consists of Dulbecco’s modified Eagle’s medium/Nutrient Mixture F-12 Ham (3:1) supplemented with 1% penicillin and streptomycin, plus the following: 1.8 × 10^−4^ M adenine, 5 μg/mL insulin, 5 μg/mL transferrin, 2 × 10^−9^ M triiodothyronine, 1.1 × 10^−6^ M hydrocortisone, 5.5 × 10^−6^ M epinephrine, 1.64 × 10^−6^ M epidermal growth factor and 10% FBS. Following culture in the appropriate medium, the cells were deprived of serum for 24 h, then subjected to the treatments.

### 2.3. Cytotoxicity Assay

To perform the assay, the cells were grown for 24 h into 96-well microplates (2 × 10^4^ cells/well), then treated with the test substances and incubated according to the applied protocol schedule [26]. At the end of incubation, the cytotoxicity was measured by the 3-[4,5-dimethylthiazol-2-yl] -2,5-diphenyl tetrazolium bromide (MTT) assay, using an Epoch Microplate Spectrophotometer (BioTek, AHSI, Milan Italy). At least three biologic replicates, in which each concentration was tested in triplicate, were made. Comparing the number of viable cells in each treatment respect to the vehicle control allows to measure a cell viability reduction. The effect of the treatment was considered as cytotoxic when at least a 30% lowering of cell viability occurred [59].

### 2.4. Schedule of Single and Metronomic Treatments

In the single long-term schedule, confluence cells were exposed to the test substances for 24 h and 72 h (Figure S1), then the cytotoxicity was measured by the MTT assay. Under the metronomic treatment, the cells were subjected to a short and/or repeated exposure of 2 h to the test substances (Figure S2), as follow: 1) single treatment, the cells were treated for 2 h, then washed and incubated for 72 h; 2) two-repeated treatments, the cells were exposed to the test substances for 2 h, then washed and incubated for functional recovery for further 2 h. After recovering, the cells were further treated with the test substances for 2 h, washed and incubated for 72 h. Finally, the cytotoxicity was measured by the MTT assay as described above.

### 2.5. Combination Assay and Analysis of Sesquiterpene-Drug Interactions

For evaluating the chemosensitizing properties, the combination of doxorubicin and a nontoxic concentration (about the IC_10_ concentrations, at which about a 10% lowering of cell viability occurred) of β-caryophyllene was administered to cells.

The type of interaction between β-caryophyllene and doxorubicin, i.e., synergy, additivity or antagonism, was evaluated by measuring three parameters: reversal ratio value (RR), combination index (CI) and isobolographic analysis (IB) [24].

RR represents the cytotoxicity enhancement ratio and allows to quantify the efficacy increase of a drug (A) in the presence of a chemosensitizing agent (B), by relating the IC_50_ of A alone (C_A_) and that of IC_50_ of its combination with B (C_A+B_).

CI gives a quantitative measure of the interaction and is calculated as follow: (C_A,X_/IC_X,A_) + (C_B, X_/IC_X,B_), in which C_A,X_ and C_B,X_ are the concentrations of A and B at the IC_50_ value of their combination, whereas IC_X,A_ and IC_X,B_ are the IC_50_ values of the substances alone. When the CI value was equal to, less or higher than 1, the interaction between test substances was evaluated as additivity, synergism and antagonism, respectively.

The isobolographic analysis, displaying the extent of the interaction between drug A and the potential chemosensitizer B, was obtained by a two-coordinate plot, wherein the IC_50_ concentrations of A and B were plotted on the x and y axes, corresponding to (C_A, 0_) and (C_0, B_), respectively. The substance concentrations used in the combination (C_A+B_) were placed in the same plot. A nonlinear regression analysis was carried out by GraphPad Prism™ 6.00 software, in order to connect the IC_50_ value of each drug alone and that of the combination, whereas the line connecting C_A_ and C_B_ represented an additive interaction. A synergism between A and B occurred when C_A+B_ were located below the line, while the interaction was antagonistic when the values were above the line [60].

### 2.6. Intracellular Reactive Oxygen Species (ROS) Determination

To perform the assay, the cells, seeded in 6-well plates (1 × 10^6^ cells/well), were treated with the tested substances (β-caryophyllene, 50 µM; doxorubicin, 20 µM) for the required time exposure, then the ROS levels were measured in the pellets by the 2,7-dichlorofluorescein diacetate assay (DCFH-DA), as previously reported [61]. Intracellular ROS proportionally reduced DCFH-DA to the fluorophore DCF, whose fluorescence was measured at an excitation wavelength of 485 nm and emission wavelength of 528 nm by a BD Accuri™ C6 flow cytometer (BD Biosciences, Milan, Italy). In each experiment, a vehicle control, corresponding to a basal ROS level, were included too. For all the treatments, the mean DCF fluorescence of 50,000 cells was determined by a BD Accuri^TM^ C6 Software version 1.0.264.21 (BD Biosciences, Milan, Italy). 

### 2.7. Chromatographic Determination of Intracellular Glutathione Levels

Intracellular levels of reduced (GSH) and oxidized (GSSG) glutathione were measured by HPLC-UV, as previously described [62]. Briefly, after treatments with β-caryophyllene (50 µM) and doxorubicin (20 µM), the harvested cells (1 × 10^6^ cells) were suspended in 10% ice-cold TCA and centrifuged for 15 min at 9000× *g*, then supernatant was collected and GSH and GSSG measured by HPLC with UV detection at 215 nm. The separation was achieved using a InfinityLab poroshell 120 EC-C18 column (3 × 150 mm, 2.7 μm) at a flow rate of 0.8 mL/min, using the following elution gradient: 0–3 min 100% A + 0% B, 3–10 min from 100% A to 100% B. The mobile phase A contained 0.1% trifluoroacetic acid in water, whereas mobile phase B was 0.1% trifluoroacetic acid in water/acetonitrile (93:7). Under our chromatographic conditions, retention times of GSH and GSSG were 2.58 min and 7.01 min, respectively.

### 2.8. Cell Cycle Analysis

Analysis of cell cycle progression was performed by applying a one parameter flow cytometric method, in which cell cycle distribution was evaluated on the basis of DNA content. Briefly, after treatments, the harvested cells (about 1 × 10^6^ cells) were fixed in ice cold 70% ethanol and stored for at least 2 h at 4 °C. Thereafter, the ethanol-suspended cells were separated by centrifugation, rinsed in PBS, then resuspended in a propidium iodide (PI) staining solution, containing 0.1% *v*/*v* Triton X-100, 10 μg/mL PI and 100 μg/mL DNase-free RNase A in PBS and stored at 4 °C in the dark for at least 30 min. For each sample, the mean fluorescence of PI (maximum excitation of PI bound to DNA at 536 nm; emission at 617 nm) in 50,000 cells was measured using a BD Accuri^TM^ C6 flow cytometer FL2 channel (BD Biosciences, Milan, Italy) and deconvolution of the DNA content frequency histograms, to estimate the proportions of cells in the respective phases of the cycle, was made by Modfit 4.1 software (Verity Software House, Topsham, ME, USA).

### 2.9. Detection of Phosphorylated Histone H2AX and STAT3 by Western Blotting Analysis

To perform the analysis, the cells were seeded in 6-well plates, then subjected to the treatment with β-caryophyllene (50 µM) and doxorubicin (20 µM), alone and in combination, for the required time exposure, then harvested by centrifugation and washed in PBS. A lysis buffer, containing SDS (2% *w*/*w*), Tris-hydrochloride (20 mM; pH 7.4), urea (2 M), glycerol (10% *w*/*w*), sodium orthovanadate (2 mM), DTT (10 mM) and a protease inhibitors cocktail (1:100 dilution) was used to achieve the cell protein separation. The separated proteins were resolved by SDS-PAGE 10% TGX FastCast™ Acrylamide gel (BioRad, Segrate, Italy) and transferred on PVDF membranes (BioRad, Segrate, Italy) by using a Trans-Blot^®^ Turbo™ Transfer System (BioRad, Segrate, Italy). The membranes were blocked with a 0.2% *w*/*v* I-block (Thermo Fisher Scientific, Rodano, Italy) in Tris-buffered saline containing 0.05% Tween-20 (TBS-T), then stained with the desired primary antibodies. Particularly, to detect the phosphorylated histone H2A variant H2AX at serine 139, the cells were incubated overnight with the anti-phospho-histone H2AX (Ser 139) antibody (rabbit antibody; SC-101696 from Santa Cruz Biotechnology), while phosphorylation of STAT3 on tyrosine 705 residue was highlighted after 1 h incubation with the anti-phospho-STAT3 (Tyr705) antibody (rabbit antibody; 9145S from Cell Signaling Technology, EuroClone, Pero, Italy). After rinsing three times in TBS-T to remove unbound primary antibody, the membranes were incubated for an additional hour with the appropriate horseradish peroxidase- or alkaline phosphatase-conjugated secondary antibody (Jackson ImmunoResearch, Pero, Italy). The peroxidase signal was detected using a ECL Fast Femto reagent (Immunological Science, Roma, Italy) and acquired by a Molecular Imager^®^ ChemiDoc™ MP System (Bio-Rad, Segrate, Italy), while the intensity of protein bands was quantified by the ImageJ software (ImageJ 1.52n, National Institutes of Health, Bethesda, MD, USA). The alkaline phosphatase signal was detected with BCIP/NBT reagents (Carl Roth, Milano, Italy, CAS No. 298-83-9 and 6578-06-9). β-actin (total extracts) was used as normalization protein for phospho(Ser 139)-histone H2AX, whereas phospho(Tyr705)-STAT3 was normalized against total STAT3 levels (anti-STAT3 total mouse antibody; 9139S from Cell Signaling Technology, EuroClone, Pero, Italy). Each experiment was performed at least in three replicates.

### 2.10. Immunofluorescence

Immunofluorescence analysis of phosphorylated STAT3 on tyrosine 705 residue was performed according to previous published method [63]. Briefly, the cells were seeded on coverslip in a six-well plate and allowed to adhere overnight, then were subjected to the treatments for the required time exposure. After treatment, the cells were fixed in 4% paraformaldehyde, washed in PBS-T and incubated in 4% bovine serum albumin (BSA) and PBS + Tween 20 (PBS-T), then further incubated with the phospho(Tyr705)-STAT3 primary antibody (9132S from Cell Signaling Technology, EuroClone, Pero, Italy) for 1 h at room temperature (RT). After washing in PBS-T, the cells were placed in the specific Alexa Fluor 488 secondary antibody (Invitrogen, Thermo Fisher Scientific, Milan, Italy) for 45 min in a dark room at RT and rinsed with PBS-T, then a coverslip was put onto slide with a drop of DAPI. Slides were examined to analyze the expression and the possible translocation of STAT3 in a coded fashion by Leica Microsystems DM 4500 B light and fluorescence microscopy (Weltzlar, Germany) equipped with a JenoptikProg-Res-C10 Plus Videocam (Jena, Germany).

A semiquantitative analysis of the phospho(Tyr705)-STAT3 rate was made (four fields for each treatment) according to a previous published grading system [64], as follow: negative, < 5%; +/−, 6–10%; +, 11–30%; ++, 31–60%; +++, > 61%.

### 2.11. Apoptosis Detection

Apoptosis extent was evaluated by Annexin-V staining (Annexin V Apoptosis detection kit, Santa Cruz Biotechnology, DBA, Milan, Italy), which is known to specifically binds phosphatidylserine, a membrane protein which is normally embedded in the inner leaflet of plasma membrane and externalized during apoptosis, as a result of the membrane changes. Therefore, apoptotic cells can be directly detected through their staining with the fluorochrome-conjugated Annexin V. The analysis was performed by both fluorescence microscopy and flow cytometry in order to both visualize the event and to achieve a quick and accurate quantification of apoptotic cells.

For flow cytometry analysis, the cells (1 × 10^6^ cells) were grown in 6-well plates, treated with β-caryophyllene (50 µM) and doxorubicin (20 µM), alone and in combination for the required time exposure, then collected by trypsinization and resuspended in PBS in the presence of the fluorochrome Annexin-V-Cy3 (4 μg/mL in cell suspension). In order to detect viable cells, the nonfluorescent probe carboxyfluorescein diacetate (CFDA; 2 μg/mL in cell suspension), hydrolyzed in living cells by esterases to its fluorescent carboxyfluorescein metabolite (CF), was included too. For each sample, the mean fluorescence of Annexin-V-Cy3 (excitation/emission at 543/570 nm, respectively) and CF (excitation/emission at 492/514 nm, respectively) in 50,000 cells was measured using a BD Accuri^TM^ C6 flow cytometer, FL-2 and FL-1 channels respectively (BD Biosciences, Milan, Italy). Although standard flow cytometry apoptosis assays use Annexin V-FITC, we chosen the conjugate with Cy3 in order to avoid the overlapping in the excitation wavelength of annexin V-FITC and CF, which could interfere with the analysis. Cells undergoing apoptosis were also sorted by their typical forward- and side scatter (FSC-SSC) pattern, i.e., increased SSC and decreased FSC, respect to the viable cells. Multiparameter analysis and gating of forward and side scatter as well as fluorescence detection were performed using a BD Accuri^TM^ C6 Software version 1.0.264.21 (BD Biosciences, Milan, Italy).

For the fluorescence microscopy analysis, 1 × 10^5^ cells were grown on coverslip in 6-well plates, treated with the test substances, then gently washed with the assay buffer, incubated for 15 min with Annexin-V-FITC working solution at RT in the dark. Thereafter, cells were rinsed in PBS, then covered with a glass coverslip with a drop of DAPI. In the end, slides were visualized under a Fluorescence Microscope (Leica Microsystems DM 4500 B Weltzlar, Germany), equipped with a JenoptikProg-Res-C10 Plus Videocam (Jena, Germany). To distinguish apoptosis from necrosis and to show membrane integrity after Annexin-V binding to cells, trypan blue exclusion test was employed in parallel. The apoptosis was semiquantitatively rated (four fields for each treatment) by applying a previous published grading system [64], as follow: negative, < 5%; +/−, 6–10%; +, 11–30%; ++, 31–60%; +++, > 61%.

### 2.12. Statistical Analysis

GraphPad Prism TM (Version 6.00) software (GraphPad Software, San Diego, CA, USA) was used for data analysis. Data were displayed as the mean ± SE (standard error) of at least two biologic replicates in which each treatment was tested in duplicate. The level of significance of the response with respect to control was evaluated by one-way analysis of variance (one-way ANOVA), followed by Dunnett’s Multiple Comparison Post Test: a *p* < 0.05 were considered as significant. The concentration–response curves were obtained by nonlinear regression, using the “Hill equation”: E = E_max_/ [1 + (10^LogEC50^/A)^HillSlope^], where E is the effect at a given concentration of the substance, E_max_ is the maximum activity, IC_50_ is the concentration that produces a 50% of the inhibitory response, A is the substance concentration, HillSlope is the curve slope.

## 3. Results

### 3.1. Cytotoxicity of β-Caryophyllene in Combination with Doxorubicin in Mz-ChA-1 Cholangiocarcinoma Cells and H69 Cholangiocytes

Cytotoxicity of the tested compounds, alone and in combination, was evaluated applying both long-term protocols of 24 h and 72 h (Figure S1) and an in vitro metronomic schedule, characterized by a single and repeated exposure of 2 h (Figure S2), as previously reported by Di Sotto et al. [26]. Preliminarily, the cytotoxicity of the natural sesquiterpene β-caryophyllene was assessed under the scheduled exposures, in order to find the suitable concentration to be used in the combination experiments.

**Figure 2:**
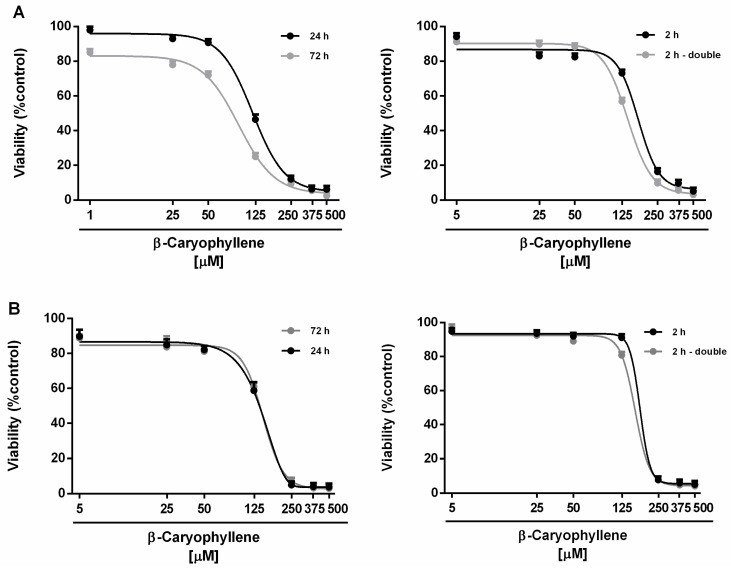
Cytotoxicity of β-caryophyllene in Mz-ChA-1 cholangiocarcinoma cells (**A**) and H69 cholangiocytes (**B**) under both the single long-term exposures of 24 h and 72 h and the metronomic schedule. In the last protocol, the cells were subjected to a single and/or double repeated short exposure of 2 h followed by a recovery time of 72 h. Data are expressed as mean ± SE (standard error) of at least two experiments in which each treatment was tested at least in triplicate (*n* = 6).

In the range of the tested concentrations (i.e., 5–500 μM corresponding to 1–100 μg/mL), β-caryophyllene produced slight cytotoxic effects in all the experimental schedules and in both cell lines (Figure 2). The cytotoxicity power of β-caryophyllene under the metronomic treatments resulted lower than that found after the long-term exposures in both cell lines. Indeed, the sesquiterpene produced early toxicity signs (about 20% inhibition of cell viability vs. control) in Mz-ChA-1 cells at concentrations higher than 50 μM after the long-term exposures of 24 h and 72 h, whereas it resulted nontoxic up to 125 μM under the metronomic schedule (Figure 2A).

In noncancerous H69 cholangiocytes, a 125 μM concentration of β-caryophyllene produced significant cytotoxic effects (about 40% inhibition of cell viability vs. control) after 24 h and 72 h, without affecting cell proliferation under the metronomic schedules (Figure 2B). Comparing the IC_50_ values, β-caryophyllene was 1.4- to 2-fold more toxic in Mz-ChA-1 cells after 72 h exposure with respect to the other schedules. Conversely, the long-term exposure treatment slightly increased its cytotoxicity in H69 cholangiocytes (maximum increase of about 1.2-fold) compared the metronomic treatments (Table 1). On the basis of these data, the concentration of 50 μM of β-caryophyllene, producing a slight or null lowering (lower than 20% reduction) of cell viability in Mz-ChA-1 and H69 cells, was chosen for the combination experiments with doxorubicin.

**Figure 3:**
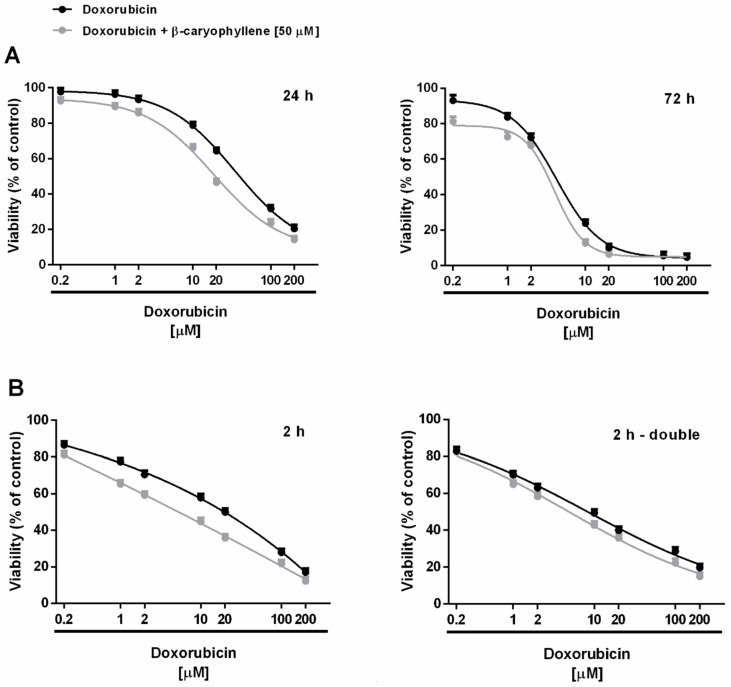
Cytotoxicity of doxorubicin and its combination with β-caryophyllene in Mz-ChA-1 cholangiocarcinoma cells. (**A**) Single long-term exposures of 24 h and 72 h. (**B**) Metronomic schedule: the cells were subjected to a single and/or double repeated short exposure of 2 h followed by a recovery time of 72 h. Data are expressed as mean ± SE (standard error) of at least two experiments in which each treatment was tested at least in triplicate (*n* = 6).

Under our experimental conditions, doxorubicin (concentration range of 0.2–200 μM corresponding to 0.1–100 μg/mL) exerted early signs of toxicity (about 20% inhibition of cell viability) at 10 µM after the long-term exposure of 24 h, achieving the maximum inhibition of about 80% at the highest tested concentration of 200 µM (Figure 3A). Cytotoxicity of doxorubicin was significantly increased by time exposure, particularly at low concentrations. Indeed, after 72 h, about a 20% inhibition of cell viability occurred already at the concentration of 1 µM, which was nontoxic under the 24 h exposure protocol (Figure 3A). The anticancer drug produced a progressive increase in the cytotoxic effect, achieving about an 80% inhibition at the concentration of 10 µM. Comparing the IC_50_ values, the long-term exposure of 72 h increased the doxorubicin potency by at least 7-fold with respect to 24 h (Table 2).

Applying the metronomic schedule, wherein the anticancer drug was administered as a short and/or double repeated treatment of 2 h followed by an extended cell recovery time, doxorubicin showed a cytotoxic profile in Mz-ChA-1 cells similar to that found after 24 h exposure (Figure 3B). Indeed, at least a 35% inhibition of cell viability was achieved at 10 μM doxorubicin after 2 h exposure protocol, with early toxicity signs (< 20% cell viability reduction vs. control) at lower concentrations, despite a 10% cytotoxicity increase produced by the double repeated exposure; a maximum 80% inhibition was achieved under both the metronomic schedules at 100 μM doxorubicin (Figure 3B).

Accordingly, the IC_50_ values of doxorubicin resulted lowered by about 1.4- and 2.4-fold when administered under the metronomic single and double repeated exposures compared the 24 h protocol, respectively (Table 2). Doxorubicin cytotoxicity was also increased after a double short exposure with respect to a single 2 h treatment, as highlighted by a 1.7-fold reduction of the IC_50_ value (Table 2). However, the anticancer power of doxorubicin under metronomic conditions was 3- to 5-fold lower than that achieved after a long-term exposure of 72 h (Table 2).

When doxorubicin was tested in combination with the chemosensitizing concentration (50 μM) of β-caryophyllene, its cytotoxicity power resulted enhanced in all the exposure schedule, except for the long-term exposure of 72 h, wherein a slight cytotoxicity increase was found only at the lower concentrations of 0.2 and 1 μM, without affecting significantly the IC_50_ value (Table 2).

Particularly, the IC_50_ value of doxorubicin in combination with β-caryophyllene was found reduced by 2- to 2.5-fold after a single long-term exposure of 24 h and under the metronomic conditions compared the anticancer drug alone (Table 2). On the basis of the obtained results, β-caryophyllene displayed chemosensitizing effects in combination with doxorubicin especially after a single treatment of 24 h and a single or double short metronomic exposure.

**Figure 4:**
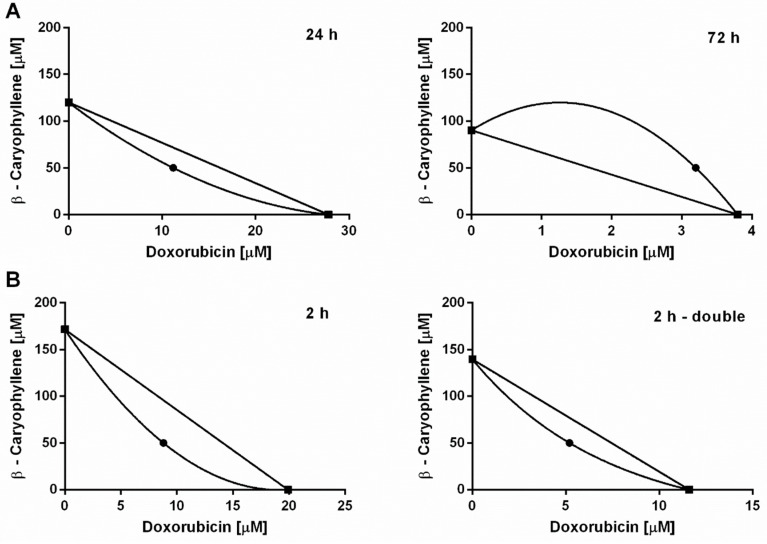
Isobolographic analysis of the cytotoxicity produced by doxorubicin in combination with β-caryophyllene at the chemosensitizing concentrations of 50 µM in Mz-ChA-1 cholangiocarcinoma cells. (**A**) Long-term exposure of 24 h and 72 h. (**B**) Single and double repeated metronomic exposure of 2 h.

According to Di Giacomo et al. [60], the interaction nature between doxorubicin and β-caryophyllene was also evaluated by means of the combination index (CI) and the isobolographic analysis (Figure 4). A lower than 1 value of CI highlights a synergistic interaction, while additivity occurs when this value is equal to 1; conversely, a higher than 1 CI denotes an antagonism. Under our experimental conditions, the combination of doxorubicin plus β-caryophyllene determined CI values of 0.82 and 1.44 after the long-term exposures of 24 h and 72 h, while CI values of 0.73 and 0.81 after a single and double repeated short metronomic exposures, respectively.

The obtained results highlighted that the chemosensitizing effects of β-caryophyllene in combination with doxorubicin were mainly ascribable to synergistic mechanisms of interaction. Accordingly, the isobologram analysis displayed a prevailing synergism between the test substances, being the point corresponding to the IC_50_ of β-caryophyllene and doxorubicin combination located below the line connecting the IC_50_ concentrations of the test substances alone (Figure 4A,B). Under a long-term exposure of 72 h, wherein the chemosensitization was lacking, both CI and isobolographic analysis displayed an antagonistic interaction between the sesquiterpene and anticancer drug (Figure 4A).

**Figure 5:**
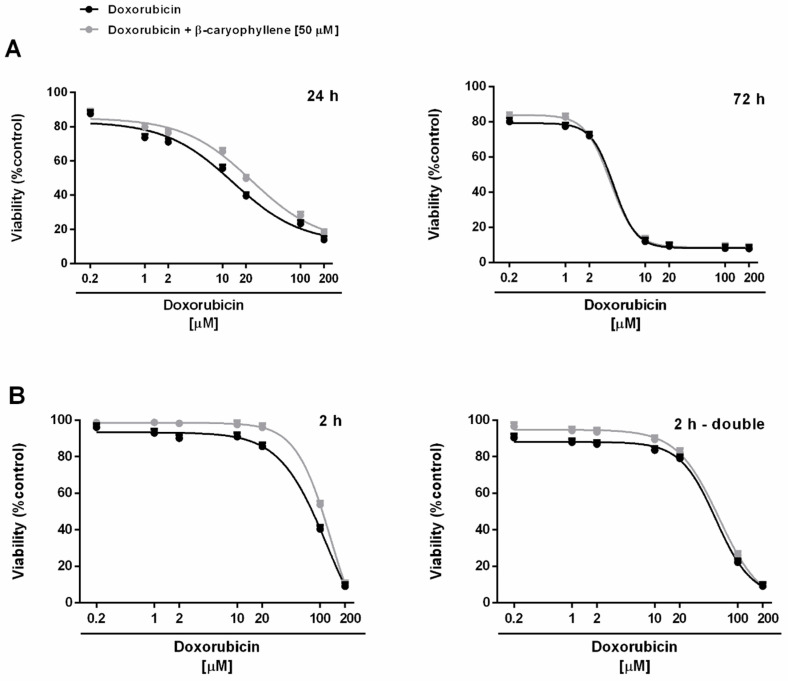
Cytotoxicity of doxorubicin and its combination with β-caryophyllene in H69 noncancerous cholangiocytes. (**A**) Single long-term exposures of 24 h and 72 h. (**B**) Metronomic schedule: the cells were subjected to a single and/or double repeated short exposure of 2 h followed by a recovery time of 72 h. Data are expressed as mean ± SE (standard error) of at least two experiments in which each treatment was tested at least in triplicate (*n* = 6).

The cytotoxic effects produced by the combination of doxorubicin and β-caryophyllene was also evaluated in noncancerous H69 cholangiocytes, applying both the long-term and metronomic schedules. After a long-term exposure of 24 h, doxorubicin exhibited early toxicity signs (about 40% inhibition of cell viability) at 10 µM, reaching the maximum inhibition of cell viability (about 80% reduction) at the highest tested concentration of 200 µM (Figure 5A). Administering the anticancer drug for a prolonged exposure of 72 h, 10 µM doxorubicin produced a maximal cytotoxicity of about 80%, resulting 2-fold more toxic than the 24 h protocol. Conversely, the low drug concentrations only slightly reduced the viability of H69 cholangiocytes, with respect to the vehicle, after both 24 and 72 h long-term exposures (Figure 5A). Comparing the IC_50_ values, doxorubicin resulted about 3-fold more toxic after 72 h with respect to 24 h (Table 2).

Under the metronomic schedules, doxorubicin was found nontoxic up to 20 μM in H69 cells, being about 100-fold safer than the long-term exposures of 72 h (Figure 5B). Conversely, a progressive reduction of cholangiocyte viability was found at concentrations higher than 20 μM, achieving a cytotoxicity of about 60% and 70% at 100 μM after a single and double repeated 2 h exposure, respectively (Figure 5B). Comparing the IC_50_ values, under the single and double repeated metronomic treatments, doxorubicin cytotoxicity was lowered by almost 7- and 3-fold with respect to the 24 h exposure (Table 2). Conversely, a double short exposure significantly increased the doxorubicin cytotoxicity compared the single one, being the IC_50_ value reduced by 1.5-fold (Table 2).

The combination of the anticancer drug with the chemosensitizing concentration (50 μM) of β-caryophyllene further reduced the cytotoxicity of doxorubicin in H69 cholangiocytes in all the experimental schedules, except for the 72 h long-term and the double repeated metronomic exposures, wherein the effects of the combined treatment did not differ from the drug alone (Figure 5A,B). Accordingly, the IC_50_ value of doxorubicin in combination with β-caryophyllene was found increased by about 1.5- and 2-fold after the single 24 h long-term and 2 h metronomic treatments (Table 2). On the basis of the obtained results, the single exposures of 2 h and 24 h, wherein β-caryophyllene in combination with doxorubicin produced both chemosensitization in Mz-ChA-1 cells and protective effects in H69 noncancerous cholangiocytes, were chosen to study the possible involved mechanisms.

The safety of the combined treatment between the sesquiterpene and the anticancer drug was also confirmed in vivo after a single administration of β-caryophyllene (50 and 100 mg/kg b.wt./i.p. in olive oil; single dose), doxorubicin (3 mg/kg b.wt./i.p. in physiological solution; single dose) and their combination in rats (Figure S3). All the procedures were performed in accordance with the International Animal Welfare Legislation (Directive 2010/63/EU, 2010).

Doses of β-caryophyllene were selected to be about 1000- and 2000-fold higher than that used in the combination experiments in liver cancer cells, while dose of doxorubicin was in agreement with literature data [65]. The dose of 50 mg/kg b.wt./i.p. was previously used for the analog β-caryophyllene oxide [25]. Under our experimental conditions, the treatments did not induce animal death and no toxicity signs were highlighted during the observation of one week after dosing and at the necroscopic analysis (Figure S3).

### 3.2. Genoprotective Effects of β-Caryophyllene in Combination with Doxorubicin

In order to determine the possible mechanisms accounting for the chemosensitizing and protective effects of β-caryophyllene towards doxorubicin, the effect of the test substances on the genotoxic damage, in term of phosphorylation of the histone H2AX at Ser139 residue, was evaluated (γH2AX). To this end, the doxorubicin concentration of 20 µM, which induced submaximal cytotoxic effects in both Mz-ChA-1 cholangiocarcinoma cells and in H69 noncancerous cholangiocytes, was chosen to be tested in combination with 50 µM β-caryophyllene under the single exposures of 2 h and 24 h, where both synergistic and chemopreventive effects of the natural sesquiterpene were displayed.

**Figure 6:**
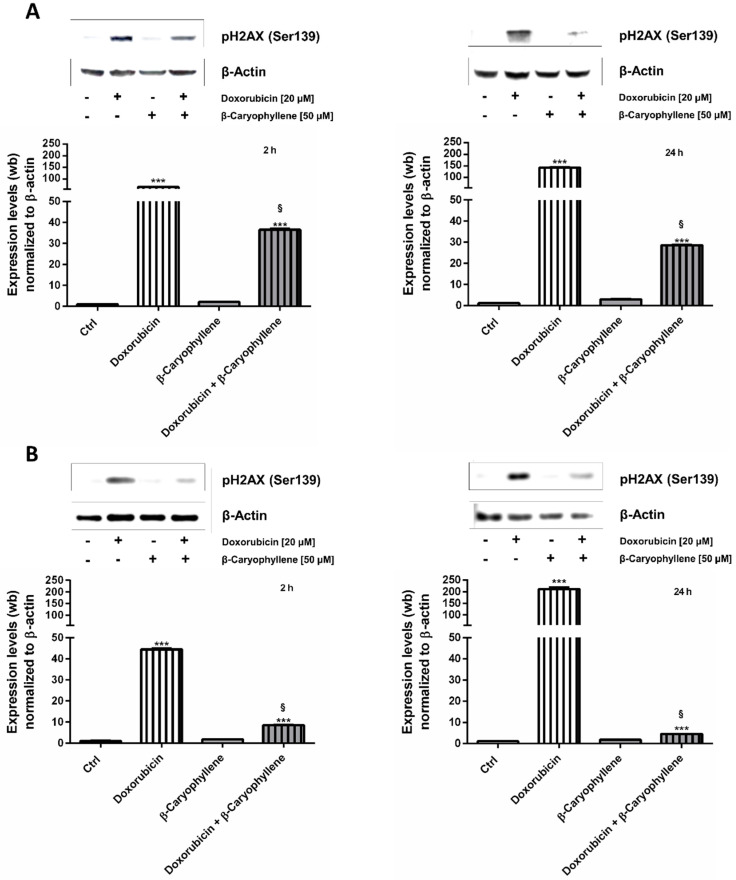
Effect of the natural sesquiterpene β-caryophyllene (50 µM), doxorubicin (20 µM) and their combination compared the control on the levels of phosphorylated H2AX at Ser139 residue (γH2AX) in Mz-ChA-1 cholangiocarcinoma cells (**A**) and H69 noncancerous cholangiocytes (**B**). The cells were treated for 2 h and 24 h, then the pellets were harvested for the western blotting analysis. For each experimental condition, the densitometric bar graph (data expressed as mean ± standard error) obtained from at least two independent replicates and a representative western blotting image, showing the expression levels of γH2AX and β-actin used as protein loading control, were displayed. ***p < 0.001 (one-way ANOVA followed by Dunnett’s multiple comparison post-test), significantly higher than the vehicle control (basal level). ^§^*p* < 0.001 (t-Student test), significantly lower than doxorubicin.

The obtained results highlighted that in spite of null effects of β-caryophyllene alone, doxorubicin markedly affected the levels of γH2AX in both Mz-ChA-1 and H69 cells after either a short exposure of 2 h and a long-term treatment of 24 h (Figure 6A,B). Particularly, in Mz-ChA-1 cells, doxorubicin produced an increase in the γH2AX levels by about 60- and 140-fold after 2 h and 24 h respectively, compared the control (Figure 6A). Similarly, in noncancerous H69 cholangiocytes about a 40- and 200-fold increase of γH2AX with respect to control was achieved after 2 h and 24 h, respectively (Figure 6B).

When doxorubicin was administered in combination with β-caryophyllene, a significant lowering of the γH2AX expression was found in all the schedules in both cancer and noncancerous cholangiocytes, although the effect resulted more pronounced in H69 cells. Indeed, despite a reduction by about 1.8- and 5-fold in Mz-ChA-1 cells under 2 h and 24 h protocols (Figure 6B), β-caryophyllene inhibited the doxorubicin-mediated γH2AX expression by at least 5- and 45-fold in H69 cells after 2 h and 24 h exposures, respectively (Figure 6B).

These data highlighted that β-caryophyllene is able to counteract DNA damage induced by doxorubicin in both cell lines, being markedly effective in noncancerous cholangiocytes.

### 3.3. Effect of β-Caryophyllene in Combination with Doxorubicin on Cell Cycle Progression

**Figure 7:**
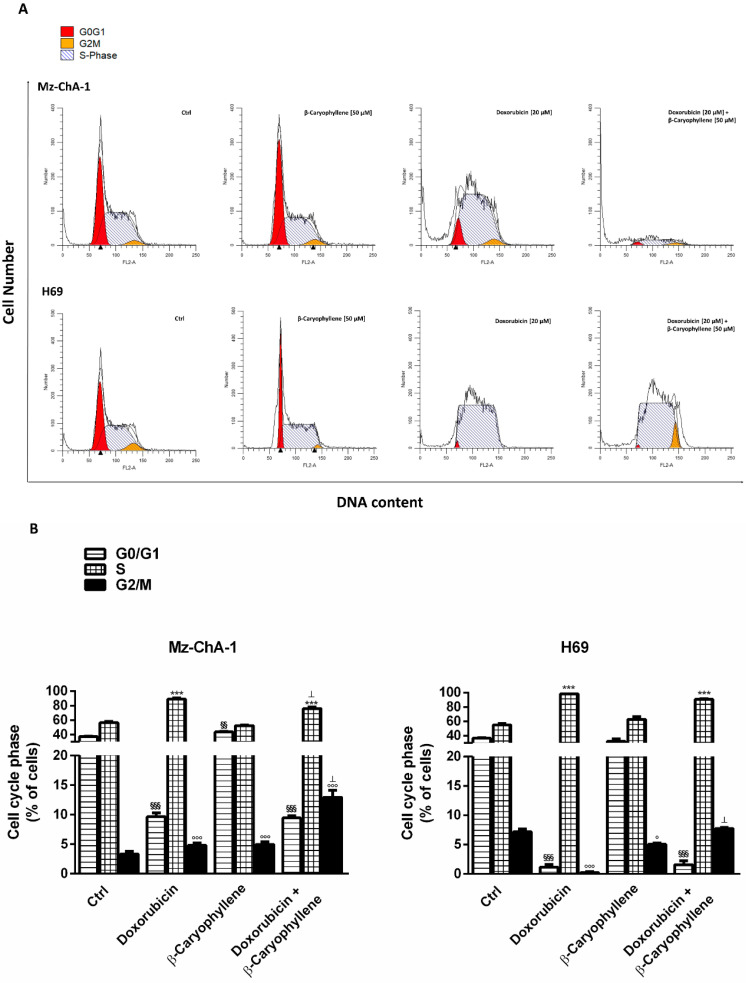
Effect of the natural sesquiterpene β-caryophyllene (50 µM), doxorubicin (20 µM) and their combination compared the control on the cell cycle progression in Mz-ChA-1 cholangiocarcinoma cells and H69 noncancerous cholangiocytes. The cells were treated for 24 h, then the pellets were harvested, fixed with 70% ethanol and stained with propidium iodide for the cytofluorimetric analysis. (**A**) Representative histograms showing the percentages of cells in different cell cycle phases after treatments in Mz-ChA-1 and H69 cells. (**B**) Bar graph analysis obtained from at least two independent replicates (data expressed as mean ± standard error). ^§§^*p* < 0.01 and ^§§§^*p* < 0.001 (one-way ANOVA followed by Dunnett’s multiple comparison post-test) denote a significant difference of G0/G1 phase in the treatments compared the control. ***p < 0.001 (one-way ANOVA followed by Dunnett’s multiple comparison post-test) denotes a significant difference of S phase in the treatments compared the control.). ^°^*p* < 0.05 and ^°°°^*p* < 0.001 (one-way ANOVA followed by Dunnett’s multiple comparison post-test) denotes a significant difference of G2/M phase in the treatments compared the control. ^⊥^*p* < 0.001 (t-Student test) denotes a significant difference with respect to doxorubicin.

In the attempt to evaluate if an interference with the cell cycle progression could be involved in the chemopreventive and chemosensitizing effects of β-caryophyllene, a flow cytometric measure of cell cycle phases in all the experimental conditions was made. The obtained results highlighted that the treatment schedules did not affected significantly the cell cycle profiles, being the effects of the treatments similar after both a single short exposure of 2 h (data not shown) and a single long-term one of 24 h (Figure 7).

Particularly, as displayed by both histograms and graph bars (Figure 7A,B), the natural sesquiterpene β-caryophyllene produced a cell cycle profile similar to that of the control, with a slight increase in the G0/G1 (about 1.2-fold vs. control) and in G2/M phases (about 1.5-fold vs. control) in Mz-ChA-1 cells, despite a weak (about 1.4-fold vs. control) reduction of G2/M phase in H69 cholangiocytes.

As expected, doxorubicin significantly enhanced the cell accumulation in S-phase in both the cell lines (about a 1.6- and 1.8-fold increase in Mz-ChA-1 and H69 compared the control), with a lower increase of G2/M phase in Mz-ChA-1 cells (about 1.4-fold vs. control); conversely, the anticancer drug markedly lowered the G0/G1 phase, achieving a reduction by at least 4 and 30-fold in Mz-ChA-1 and H69 cells, respectively (Figure 7).

Combining β-caryophyllene and doxorubicin retained the cell cycle profile of the anticancer drug, except for a slight reduction (1.1-fold vs. doxorubicin) of S-phase in Mz-ChA-1 cells and a marked increase of G2/M phase in both cell lines (Figure 7). Particularly, G2/M phase was enhanced by almost 3-fold with respect to doxorubicin alone in Mz-ChA-1 cells, being also 3-fold higher than the control. In the H69 cells, a significant 4-fold enrichment of cells in G2/M phase compared doxorubicin also occurred, restoring the same level in the control. The obtained results highlighted that β-caryophyllene contributed to the S-phase cell cycle arrest induced by doxorubicin and also blocked cells in G2/M phase. Comparing cholangiocarcinoma and noncancerous cholangiocytes, we also found that despite similar levels of both G0/G1 and S phases, G2/M resulted lowered by at least 2-fold in Mz-ChA-1 cancer cells with respect to the H69 cholangiocytes (Figure 7), thus suggesting that a lowered G2/M phase arrest can support the progression of cholangiocarcinoma cells.

### 3.4. Effect of β-Caryophyllene on the Intracellular Oxidative Stress

**Figure 8:**
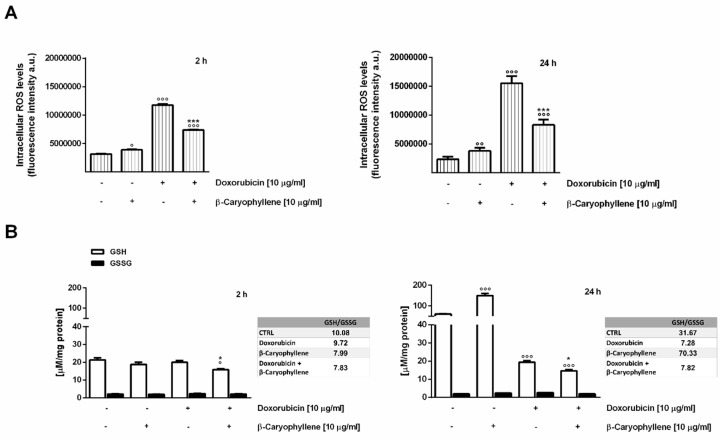
Effect of the natural sesquiterpene β-caryophyllene (50 µM), doxorubicin (20 µM) and their combination compared the control on the intracellular oxidative stress in Mz-ChA-1 cholangiocarcinoma cells. The cells were treated for 2 h and 24 h, then the pellets were harvested for the subsequent analysis. (**A**) Bar graphs representing the levels of reactive oxygen species (ROS) as detected by the 2,7-dichlorofluorescein diacetate (DCFH-DA) assay. Data are expressed as mean ± standard error of at least two independent replicates. (**B**) Bar graphs representing the levels of GSH (reduced glutathione) and GSSG (oxidized glutathione) as revealed by HPLC analysis. Data are expressed as mean ± standard error of at least two independent replicates. ^°^*p* < 0.05, ^°°^*p* < 0.01 and ^°°°^*p* < 0.001 (one-way ANOVA followed by Dunnett’s multiple comparison post-test) denote a significant difference of the treatments compared the control. *p < 0.05 and ***p < 0.001 (t-Student test) denote a significant difference with respect to doxorubicin.

The effect induced by the combination of doxorubicin and β-caryophyllene on the intracellular oxidative stress was evaluated after both 2 h and 24 h exposures in both the cell lines, by measuring the levels of reactive oxygen species (ROS) by flow cytometry and those of glutathione defenses by chromatographic detection. In Mz-ChA-1 neoplastic cholangiocytes, the natural sesquiterpene induced a slight but significant increase (about 1.6 and 1.2-fold after 2 h and 24 h treatment) of ROS levels (Figure 8A). Also, the GSH levels resulted significantly (about 2.5-fold vs. control) increased after 24 h exposure, without changes in GSSG (Figure 8B).

The anticancer drug markedly enhanced the ROS levels, achieving almost a 7- and 4-fold increase with respect to the control after 2 h and 24 h of treatment, respectively (Figure 8A). GSH resulted not significantly decreased by the drug after 2 h, whereas a reduction by at least 3-fold was found after 24 h exposure, without changes in the GSSG amount (Figure 8B).

When the sesquiterpene was assessed in combination with doxorubicin, ROS levels were found reduced by almost 2-fold under both time schedules and a further lowering (about a 1.3- and 4-fold reduction after 2 h and 24 h exposure vs. doxorubicin) of GSH amount with respect to doxorubicin occurred (Figure 8A,B). Accordingly, GSH/GSSG ratio resulted lowered by 1.3- and 4 -fold in the presence of doxorubicin or its combination with β-caryophyllene after 2 h and 24 treatments; conversely, β-caryophyllene alone was able to increase this ratio by about 2-fold.

**Figure 9:**
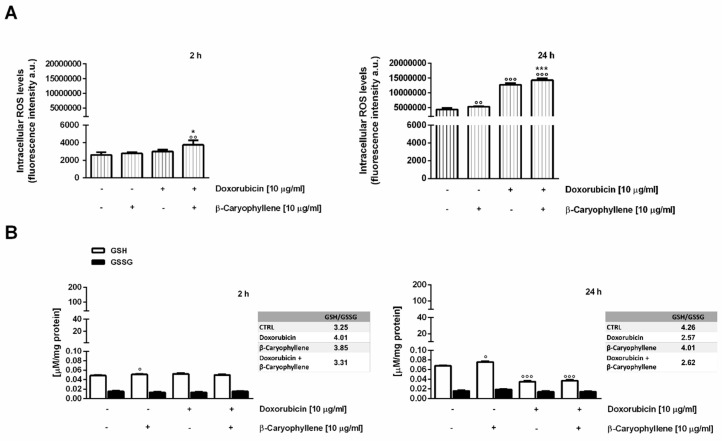
Effect of the natural sesquiterpene β-caryophyllene (50 µM), doxorubicin (20 µM) and their combination compared the control on the intracellular oxidative stress in H69 noncancerous cholangiocytes. The cells were treated for 2 h and 24 h, then the pellets were harvested for the subsequent analysis. (**A**) Bar graphs representing the levels of reactive oxygen species (ROS) as detected by the 2,7-dichlorofluorescein diacetate (DCFH-DA) assay. Data are expressed as mean ± standard error of at least two independent replicates. (**B**) Bar graphs representing the levels of GSH (reduced glutathione) and GSSG (oxidized glutathione) as revealed by HPLC analysis. Data are expressed as mean ± standard error of at least two independent replicates. ^°^*p* < 0.05, ^°°^*p* < 0.01 and ^°°°^*p* < 0.001 (one-way ANOVA followed by Dunnett’s multiple comparison post-test) denote a significant difference of the treatments compared the control. *p < 0.05 and ***p < 0.001 (t-Student test) denote a significant difference with respect to doxorubicin.

The ability of doxorubicin and β-caryophyllene to affect the redox homeostasis was also assessed in noncancerous H69 cholangiocytes, under the same experimental conditions. After a short exposure of 2 h, ROS levels resulted not significantly enhanced by treatments, except for a 1.5- and 1.3-fold increase induced by the combination of the natural sesquiterpenes and doxorubicin compared to the control and doxorubicin, respectively (Figure 9A). Accordingly, the treatments only slightly affected GSH and GSSG levels (Figure 9B): a 1.2-fold increase in GSH/GSSG ratio was found in the presence of the test substances alone, but not with their combination.

When assessed under a long-term exposure of 24 h, despite a weak 1.3-fold enhancement in ROS amount induced by β-caryophyllene compared the control, doxorubicin and its combination with β-caryophyllene markedly increased the ROS levels (at least 3-fold higher than the control) (Figure 9A). As for the 2 h protocol, GSH resulted enhanced by almost 1.5-fold by β-caryophyllene after 24 h, whereas depleted by almost 2-fold by doxorubicin and its combination with β-caryophyllene (Figure 9B). Accordingly, the amount of GSSG was slightly increased in the presence of the only β-caryophyllene, while slightly but not significantly reduced by doxorubicin and its combination with the sesquiterpene (Figure 9B). The basal GSH/GSSG ratio was retained in the presence of β-caryophyllene, while lowered by almost 1.6-fold with doxorubicin and the combination, thus suggesting that an impairment of cell defenses, likely due to the increased ROS levels, occurred (Figure 9A,B).

### 3.5. Effect of β-Caryophyllene in Combination with Doxorubicin on Cell Apoptosis

As for the other parameters, apoptosis was measured in both the cell lines after both 2 h and 24 h exposure to doxorubicin, β-caryophyllene and their combination; also, viable cells were detected in the same analysis by staining them with specific probe for apoptotic and viable cells. Particularly, apoptosis was detected by using a fluorochrome-conjugated annexin V that specifically binds to phosphatidylserine, a cell protein that is translocated from the cytoplasmic face of the plasma membrane to the cell surface during early apoptosis, thus being a signal of a biomembrane phospholipid asymmetry loss. Furthermore, the nonfluorescent carboxyfluorescein diacetate (CFDA) probe was employed to distinguish viable cells, being it able to be taken up by cells, then hydrolyzed by esterases, producing a fluorescent 6-carboxyfluorescein (6-CF) metabolite.

Also, live and apoptotic cells were selected by their typical cell size and granularity, measured through the forward light scatter (FSC) and side scatter (SSC). Particularly, increased SSC and decreased FSC occurred during apoptosis, due to the cell shrinkage and mitochondrial swelling and to the nuclear condensation and fragmentation, respectively. Conversely, excluding events with low FSC and high SSC, viable cells were sorted.

**Figure 10:**
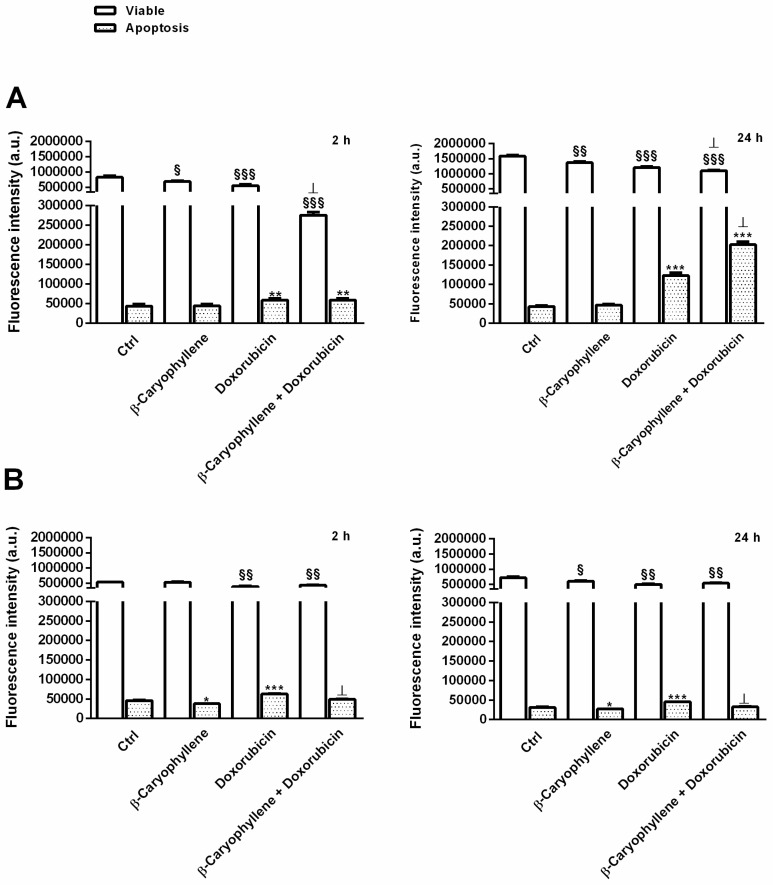
Bar graphs (data expressed as mean ± standard error at least two independent replicates) of apoptosis induced by the natural sesquiterpene β-caryophyllene (50 µM), doxorubicin (20 µM) and their combination compared the control in Mz-ChA-1 cholangiocarcinoma cells (**A**) and H69 noncancerous cholangiocytes (**B**). The cells were treated for 2 h and 24 h, then the pellets were harvested and stained with Annexin-V-Cy3 and carboxyfluorescein diacetate (CFDA) for the cytofluorimetric analysis of apoptotic and viable cells (FL-2 for annexin and FL-1 for CF; BD AccuriTM C6 flow cytometer). Cells undergoing apoptosis were sorted by their typical forward- and side scatter (FSC-SSC) pattern, i.e., increased SSC and decreased FSC, respect to the viable cells. ^§^*p* < 0.05, ^§§^*p* < 0.01 and ^§§§^*p* < 0.001 (one-way ANOVA followed by Dunnett’s multiple comparison post-test) denote a significant difference in the viable cells of the treatments compared the control. *p < 0.05, **p < 0.01 and ***p < 0.001 (one-way ANOVA followed by Dunnett’s multiple comparison post-test) denote a significant difference in the apoptotic cells of the treatments compared the control. ^⊥^*p* < 0.01 (t-Student test) denotes a significant difference with respect to doxorubicin.

Under our experimental condition, as displayed by the CF fluorescence intensity detected at FL-1 channel (excitation/emission at 492/514 nm, respectively), viable cells were found only slightly affected by β-caryophyllene, achieving a maximum inhibition by about 1.2-fold in both Mz-ChA-1 and H69 cells under both the short and long-term exposures of 2 h and 24 h, respectively (Figure 10A,B). Doxorubicin lowered the viable cell rate up to 1.5 and 1.4-fold in Mz-ChA-1 and H69 cells, respectively, whereas its combination with β-caryophyllene further reduced cell viability in Mz-ChA-1 cell especially after 2 h exposure (almost a 2-fold reduction), despite a slight increase in H69 cell levels with respect to the anticancer drug (Figure 10A,B).

In regard to the apoptosis extent, measured by the fluorescence intensity of annexin-V-Cy3 at FL-2 channel (excitation/emission at 543/570 nm, respectively), β-caryophyllene did not affected the basal apoptosis in Mz-ChA-1, although a slight but significant 1.2-fold lowering in H69 cell under both time exposures was registered (Figure 10A,B). As expected, apoptosis was significantly enhanced by doxorubicin, especially after a long-term exposure of 24 h: the apoptotic rate was increased by almost 1.5 and 3-fold in H69 and Mz-ChA-1 cells, respectively (Figure 10A,B). Surprisingly, the apoptosis rate induced by doxorubicin significantly decreased in combination with β-caryophyllene in H69 cholangiocytes, achieving similar levels in the control, whereas it resulted enhanced by about 1.7-fold (almost 5-fold higher than the basal rate in the control) in Mz-ChA-1 cholangiocarcinoma cells (Figure 10A,B).

**Figure 11:**
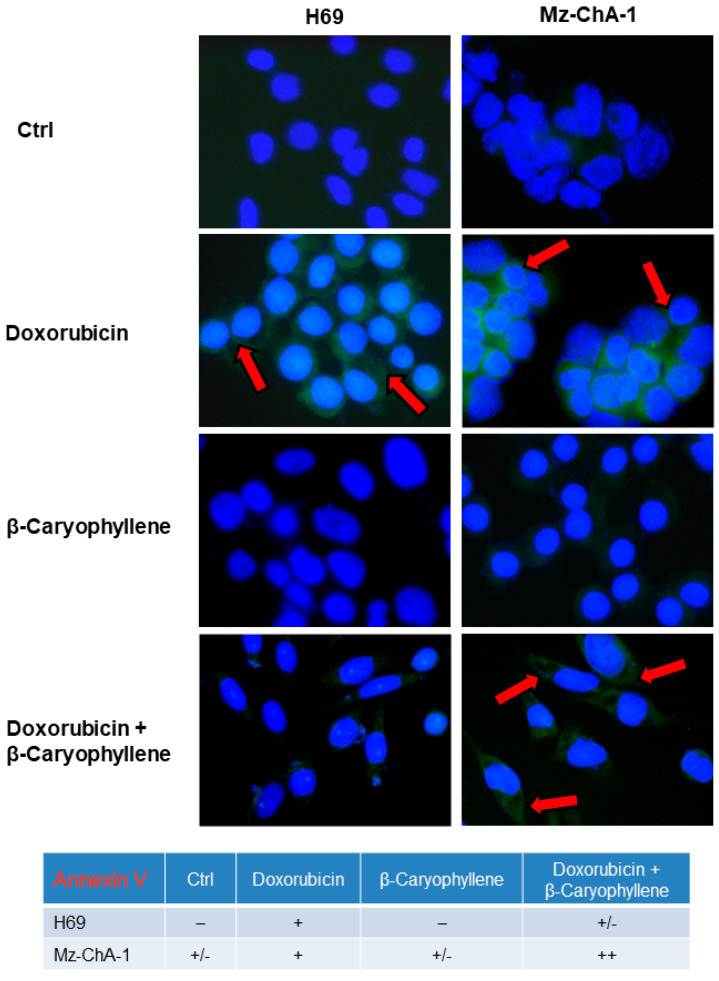
Representative immunofluorescence (IF) images and semiquantitative analysis of the apoptosis induced by the natural sesquiterpene β-caryophyllene (50 µM), doxorubicin (20 µM) and their combination compared the control in Mz-ChA-1 cholangiocarcinoma cells and H69 noncancerous cholangiocytes. After treatments of 24 h, the cells were stained with Annexin-V-FITC to assess the apoptotic rate, as shown by the red arrows. The semiquantitative analysis has been carried out (four fields for each treatment) applying a previous published grading system [64]: 0%–5% = negative; 6%–10% = +/−; 11%–30% = +; 31%–60% = ++; > 61% = +++.

These results were also confirmed by the immunofluorescence microscopy analysis (Figure 11), made by staining cells with annexin-V-FITC and by comparison with the trypan blue exclusion test to distinguish apoptosis from necrosis and to show membrane integrity. As shown in Figure 11, after treatment with the β-caryophyllene alone, the fluorescence intensity was similar to that of the control in both cell lines; particularly, a basal apoptosis was revealed only in cancer cholangiocytes. Doxorubicin induced the annexin-V binding with PS, as evidenced by the fluorescent cells detected in both H69 and Mz-ChA-1 cells, especially in the cancer cell line (Figure 11). The extent of fluorescent cells induced by the anticancer drug resulted lowered by the natural sesquiterpene in H69 cholangiocytes, while enhanced in cholangiocarcinoma cells (Figure 11).

The results were further confirmed by the scoring of the semiquantitative analysis, wherein the apoptosis rate induced by the combination of β-caryophyllene and doxorubicin in Mz-ChA-1 cells resulted to be highly positive, corresponding to a 31%–60% (++ score) level, with respect to the 11%–30% (+ score ) of doxorubicin alone, whereas it was slight positive (+/− score; 6%–10%) for the control and the only sesquiterpene (Figure 11).

### 3.6. Effect of β-Caryophyllene in Combination with Doxorubicin on the STAT3 Signaling

**Figure 12:**
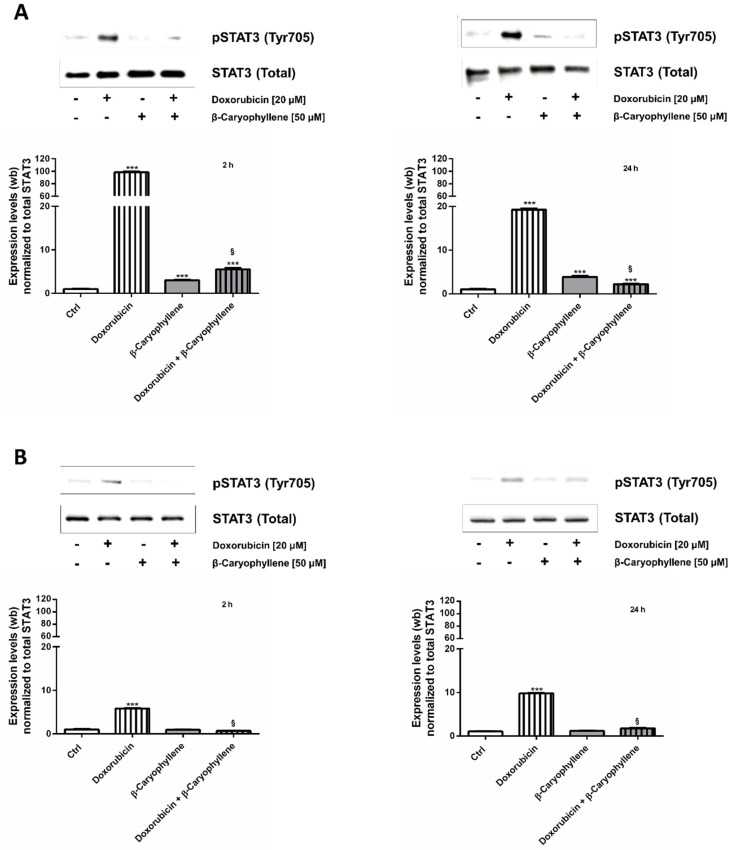
Effect of the natural sesquiterpene β-caryophyllene (50 µM), doxorubicin (20 µM) and their combination compared the control on the expression levels of phosphorylated STAT3 on tyrosine 705 residue in Mz-ChA-1 cholangiocarcinoma cells (**A**) and H69 noncancerous cholangiocytes (**B**). The cells were treated for 2 h and 24 h, then the pellets were harvested for the western blotting analysis. For each experimental condition, a representative western blotting image, showing the expression levels of the phospho(Tyr705) STAT3 and total STAT3 used as protein loading control and a densitometric bar graph analysis (data expressed as mean ± standard error) obtained from at least two independent replicates, were displayed. *** *p* < 0.001 (one-way ANOVA followed by Dunnett’s multiple comparison post-test) denotes a significant increase compared the control. ^§^ *p* < 0.001 (t-Student test) denotes a significant reduction with respect to doxorubicin.

Taking into account the widespread recognized control of STAT3 signaling on both normal and cancer cell proliferation, a possible modulation by treatments of this signaling in both Mz-ChA-1 cancer and H69 noncancerous cholangiocytes was also assessed (Figure 12).

As found at the western blotting analysis, doxorubicin markedly enhanced the phosphorylation of STAT3 at tyrosine 705 residue in Mz-ChA-1 cells, achieving almost a 100- and 20-fold increase after 2 h and 24 h treatments compared the control (Figure 12A). Likewise, the anticancer drug enhanced by almost 6 and 10-fold the basal expression of phospho(Tyr705)-STAT3 in H69 cholangiocytes after 2 h and 24 h, respectively (Figure 12B).

Interestingly, the natural sesquiterpene alone did not affected the protein expression in H69 cells, whereas it induced at least a 3-fold upregulation in neoplastic cholangiocytes under both time schedules (Figure 12A,B). Combining the sesquiterpene and doxorubicin a significant downregulation of phospho(Tyr705)-STAT3, compared the anticancer drug alone, occurred in both cell lines and in all the experimental conditions. This inhibitory effect was highly marked in Mz-ChA-1 cells, achieving a lowering by about 16 and 10-fold with respect to doxorubicin after 2 h and 24 h, respectively; a lower but significant 8- and 5-fold reduction occurred in H69 cells too (Figure 12A,B).

**Figure 13:**
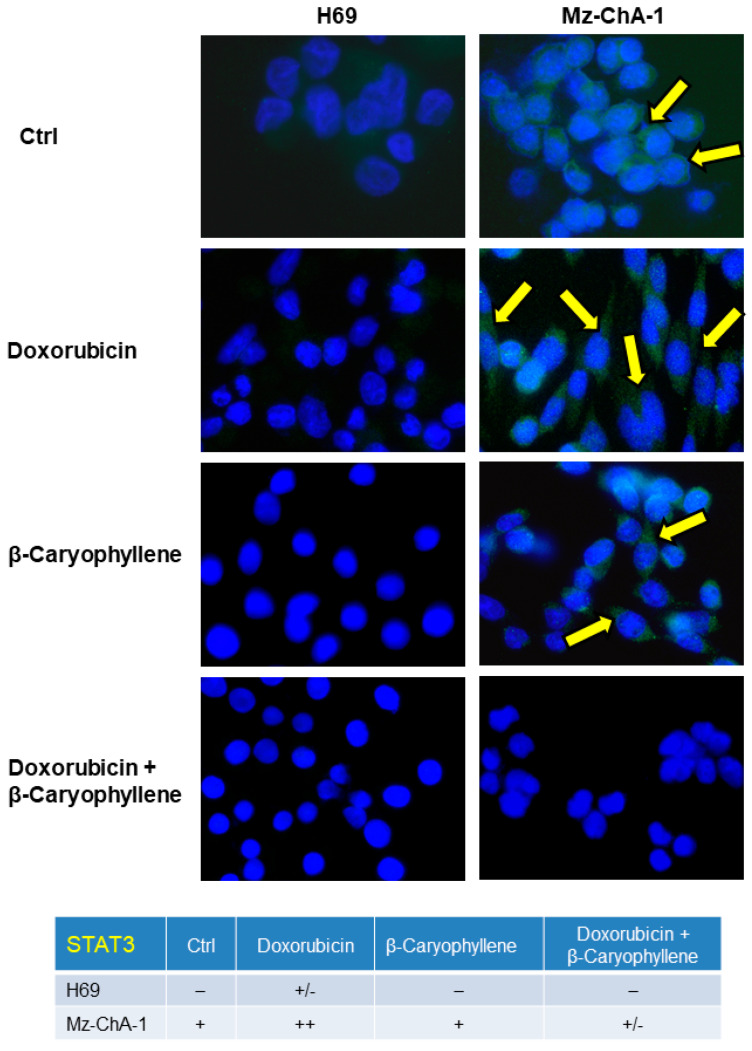
Representative immunofluorescence (IF) images and semiquantitative analysis of the phosphorylated STAT3 on tyrosine 705 residue induced by the natural sesquiterpene β-caryophyllene (50 µM), doxorubicin (20 µM) and their combination compared the control in Mz-ChA-1 cholangiocarcinoma cells and H69 noncancerous cholangiocytes. After treatments of 24 h, the cells were fixed then stained with a specific anti-phospho(Tyr705)-STAT3 primary antibody to assess the protein phosphorylation rate, as shown by the yellow arrows. The semiquantitative analysis has been carried out (four fields for each treatment) applying a previous published grading system [64]: 0%–5% = negative; 6%–10% = +/−; 11%–30% = +; 31%–60% = ++; > 61% = +++.

Accordingly, the immunofluorescence analysis displayed that phospho(Tyr705)-STAT3 fluorescence was higher in doxorubicin-treated Mz-ChA-1 cells, compared the control cells (Figure 13). By contrast, the doxorubicin-induced fluorescence was markedly downregulated in combination with β-caryophyllene, without notable effects of the sesquiterpene alone (Figure 13). Similarly, the protein resulted phosphorylated at tyrosine 705 residue by doxorubicin in H69 cholangiocytes (Figure 13), although the fluorescence intensity was lower than that displayed in Mz-ChA-1 cells. Conversely, both in the combination and in the presence of β-caryophyllene alone, the fluorescence was lacking (Figure 13), thus highlighting that the natural sesquiterpene inhibited the expression of phospho(Tyr705)-STAT3 induced by the anticancer drug. It is noteworthy that a basal phospho(Tyr705)-STAT3 was found expressed in Mz-ChA-1 cholangiocarcinoma cells compared the noncancerous H69 cholangiocytes, thus confirming that the upregulation of this signaling is a typical feature of cancer cells.

In order to assess whether the chemosensitizing effects of β-caryophyllene (50 μM) towards doxorubicin were dependent on STAT3, we also tested the substances and their combination in human prostatic PC3 cells (Figure S4), known to be lacking in the expression of STAT3 [66,67]. Under our experimental conditions, the cytotoxic effect of doxorubicin was slightly increased by β-caryophyllene, although no statistical difference was found in the IC_50_ values (48.4, C.L. 29.2–65.8 μM and 44.1, C.L. 19.8-72.1 μM for doxorubicin and doxorubicin plus β-caryophyllene, respectively) (Figure S4A). Furthermore, immunofluorescence images revealed that β-caryophyllene did not affect the doxorubicin-induced apoptosis in PC3 cells (Figure S4B). The western blotting analysis confirmed that PC3 cells did not express STAT3 (Figure S4C).

These results reinforce our hypothesis about the modulation of STAT3 signaling as one of the networking mechanisms involved in the chemopreventive and chemosensitizing effects of β-caryophyllene in combination with doxorubicin.

## 4. Discussion

Chemosensitizers are defined as low toxic agents, able to affect tumor survival and progression and to increase cancer sensitivity to pharmacotherapy by different synergistic or additive mechanisms, thereby reducing chemotherapy toxicity and occurrence of multidrug resistance [60]. Searching for chemosensitizing agents has attracted great attention by researchers due to the interesting possible application of this strategy in adjuvant chemotherapy or in sensitizing resistant cancer cells to the anticancer drugs. Recently, a complex so-called *“resistome”*, defined as the entire set of proteins contributing to the mechanisms of chemoresistance exploited by tumor to endure chemotherapeutic effects, has been characterized [68,69]. Among them, defective DNA repair pathways, altered growing signaling and an imbalance between pro-apoptotic and pro-survival factors have been found responsible for cholangiocarcinoma resistance to pharmacological treatment [7,70,71]. Therefore, targeting specific deregulated mechanisms through suitable chemosensitizers have been approached as a promising strategy to counteract CCA resistance and improve chemotherapy efficacy.

In line with this evidence, in the present study, we found that the natural chemosensitizer β-caryophyllene was able to potentiate the anticancer effects of doxorubicin in cholangiocarcinoma Mz-ChA-1 cells and also to prevent doxorubicin-toxicity in noncancerous H69 cholangiocytes, thus highlighting a possible novel strategy to achieve both chemotherapy efficacy and drug tolerability.

The doxorubicin chemosensitization by β-caryophyllene in cholangiocarcinoma cells is in agreement with our previous evidence in HepG2 [26]. Similarly, β-caryophyllene oxide produced synergistic effects towards doxorubicin and other anticancer drugs in liver, breast, leukemic and colon cancer cells [24,25,26,72,73,74]. Interestingly, doxorubicin-chemosensitization by β-caryophyllene occurred after both a long-term exposure of 24 h and under the metronomic schedules, like in HepG2 cells [26]. Conversely, a long-term exposure of 72 h negatively affected the chemosensitizing properties of β-caryophyllene, likely due to its possible biotransformation into ineffective metabolites [26,75,76]. According to the literature [26,77], the metronomic schedules increased the cytotoxicity of low dose doxorubicin in cholangiocarcinoma cells too. Therefore, combining the doxorubicin chemosensitization by β-caryophyllene and the metronomic schedule appears to be an interesting strategy to increase the drug potency and to reduce its toxicity. Further *in vivo* studies could confirm this evidence and support a future interest in pharmacological research.

In regard to the mechanisms of action, doxorubicin is known to possess a powerful cancer-killing potential, mediated by multiple cytotoxic mechanisms, involving increased intracellular oxidative stress, inhibition of topoisomerase IIα, DNA-damage and blocking of cancer cell growth, which in turn lead to destruction of cell structures and cell death [78]. Induction of ROS formation, interference with mitochondrial oxidative phosphorylation and GSH depletion represent key mechanisms of anthracycline toxicity [78,79,80].

**Figure 14:**
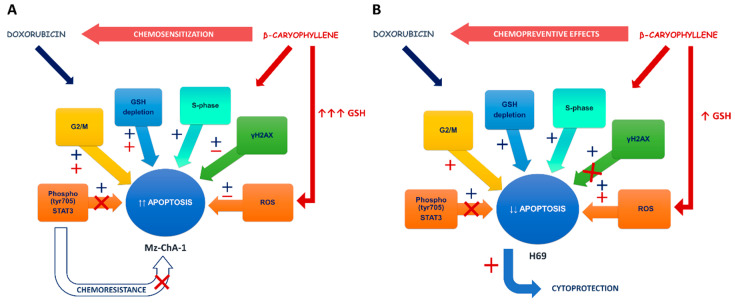
Scheme of the possible network involved in the chemosensitizing and chemopreventive effects of β-caryophyllene towards doxorubicin in Mz-ChA-1 cholangiocarcinoma cells (**A**) and in H69 noncancerous cholangiocytes (**B**). Doxorubicin-induced oxidative stress, measured by the intracellular levels of reactive oxygen species (ROS), was partly reduced in combination with β-caryophyllene in Mz-ChA-1 cells, whereas GSH defenses, markedly upregulated by the sesquiterpene alone, were drastically reduced by both doxorubicin and its combination with β-caryophyllene, without affecting GSSG amount. Conversely, in H69 cholangiocytes, despite a GSH depletion similar to doxorubicin and not correlated with an increase in GSSG, the combined treatment of doxorubicin and β-caryophyllene enhanced ROS levels with respect to both control and the anticancer drug alone, despite a slight increase of GSH by the only β-caryophyllene. γH2AX, a biomarker of doxorubicin-induced DNA-damage, resulted partly lowered by the presence of β-caryophyllene in cholangiocarcinoma cells, with an almost complete inhibition in normal cholangiocytes. The increased G2/M checkpoint phase by β-caryophyllene could allow to repair the doxorubicin-damaged DNA in H69 cholangiocytes. In both cell lines, the phosphorylation of STAT3 at tyrosine 705 site, induced by the anticancer drug, resulted markedly lowered by the natural sesquiterpene. These networking effects result in an increased apoptotic fate in cholangiocarcinoma Mz-ChA-1 cells, despite an inhibition apoptosis in H69 noncancerous cholangiocytes.

Under our experimental conditions, the behavior of doxorubicin in both cholangiocarcinoma and noncancerous cholangiocytes was in agreement with published literature, being the drug responsible for an increased oxidative stress along with a lowering of GSH, especially after a long-term exposure (Figure 14A,B). Interestingly, GSH depletion was not associated with an increase in GSSG levels, thus suggesting that reduced glutathione was exploited by cells for alternative requirements.

β-caryophyllene induced a significant ROS-increase in cancer cells and a marked upregulation of GSH defenses, especially after 24 h exposure, without affecting cell viability: this could be ascribed to a perturbation of cell biomembrane homeostasis, due to the high lipophilicity of the sesquiterpene. Indeed, β-caryophyllene is known to possess a great capacity to alter phospholipid cooperativity, membrane permeability and protein functions [45,81], which in turn can lead to cell morphology changes and increased of intracellular oxidative stress [46].

Conversely, in the presence of doxorubicin/β-caryophyllene combination, the ROS levels were partly reduced in cholangiocarcinoma cells despite a significant increase in normal cells, with a marked GSH depletion in both cell lines (Figure 14A,B).

Glutathione has been shown to act as a protective factor in both cancer and normal tissues, being able to detoxify xenobiotics and ROS species by its antioxidant power or by glutathione S-transferase (GST)-mediated phase II reactions [82].

In malignant cells, both ROS levels and GSH defenses have been found highly expressed and this redox state seems to underpin cell resistance to many stressors, among which chemotherapeutic drugs and to inhibit programmed cell death. GSH upregulation allows drug-conjugation and excretion through MRP transporters, thus leading to cancer resistance [82]. In support, a high expression of both GST and MRP pumps as well as high GSH levels represent common features of cholangiocarcinoma cells [51,53]. Particularly, high GSH content can promote tumor cell survival by inhibiting apoptosis and inducing post-translational modifications of transcription factors and proteins involved in the control of cancer progression [82].

In line with this evidence, we hypothesize that when doxorubicin enters cholangiocytes, it can be conjugated by glutathione, thus forming GSH-doxorubicin conjugates which are excreted through MRP transporters, being toxic for cells. It is known that MRP1 and MRP3 are abundantly expressed in cholangiocarcinoma [69,83,84], whereas MRP2 is typical of normal hepatocytes and cholangiocytes [85]. The constant levels of GSSG found under our experimental conditions further confirm the hypothesis that GSH expenditure was mainly due to doxorubicin detoxification, with a lower impact on ROS neutralization.

The GSH upregulation induced by β-caryophyllene in cholangiocarcinoma cells seems to be mainly exploited for doxorubicin detoxification, being unchanged the GSSG levels. Nevertheless, considering that β-caryophyllene is able to inhibit MRP pumps [25], GSH-doxorubicin conjugates can be accumulated into cells, thus leading to the activation of cell death signalings. Our hypothesis is supported by the evidence that lowering the intracellular GSH content has been found associated with increased ROS amounts and apoptosis, thus being studied for potential anti-cancer therapies [86]. Also, using inhibitors of MRP-mediated transport of GSH-conjugates has been approached as an alternative strategy to overcome multidrug resistance and resensitize cells to chemotherapy [87].

Despite the increased GSH levels in cholangiocarcinoma cells, the sesquiterpene alone slightly affected GSH in normal cholangiocytes: this could be reflected in the high ROS levels found in the combined treatment with doxorubicin, being GSH unable to neutralize the intracellular oxidative stress. Nevertheless, characterizing the true mechanisms responsible for the different behavior of β-caryophyllene in cancer and normal cholangiocytes requires further investigations.

The increased ROS levels by doxorubicin are also associated with DNA-damage, although other genotoxic mechanisms, such as a direct DNA-intercalation through the planar aromatic aglycone and a topoisomerase IIα inhibition, which result in the accumulation of double-strand DNA breaks, have been reported [10]. Double-strand DNA breaks by doxorubicin are usually correlated with a G2-phase cell cycle block, while a S-phase accumulation is associated to DNA adducts [88].

Under our experimental conditions, doxorubicin was found able to increase γH2AX levels after both a short and long-term exposure in both cell lines (Figure 14A,B): this effect was correlated with an accumulation of cholangiocarcinoma cells in both S and G2/M phases, while the S phase greatly prevailed in normal cholangiocytes. These results suggest that doxorubicin genotoxicity could be mediated by both topoisomerase II inhibition and formation of DNA-adducts in biliary cancer cells, whereas the formation of DNA-adducts could mediate drug toxicity in normal cells.

The sesquiterpene alone significantly increased the cell accumulation in G1 and G2/M phases, without affecting γH2AX levels. In the presence of β-caryophyllene, the doxorubicin-increased γH2AX levels were significantly lowered in both cell lines, although with about a 9-fold higher efficacy in H69 cells compared Mz-ChA-1 cells (Figure 14A,B). Furthermore, in both cell lines, along with a cell cycle arrest in S-phase induced by doxorubicin, β-caryophyllene significantly increased the cell percentage in G2/M phase: this point was of particular interest in H69 cholangiocytes, wherein this phase was almost lacking (Figure 14A,B).

The normal cell cycle can be modulated by many different factors, thus leading to alterations in cell proliferation which represent important features of cancer cells. Usually, after a genome damage, a DNA damage response (DDR) occurs, followed by the activation of DNA damage checkpoints (G1 and G2 phases), wherein cell cycle is blocked, thus restraining chromosome segregation until the damage could be fixed, and DNA repair systems are stimulated [89,90].

A loss of G1 checkpoint is a common feature of cancer cells and allows mutagenic replication of damaged templates and other replication defects: this failure makes cells more reliant upon the S and G2 checkpoints to prevent DNA damage-triggered cell death [91]. Also, G2/M checkpoint can fail due to the presence of unreplicated or damaged DNA [90]. Phosphorylation of H2AX at the serine residue (S139) represents one of the earliest events upon DNA double-strand breaks and is involved in DDR induction, delayed cell cycle progression and DNA repair, thus leading to cells recovering or death if damage is unrepairable [92].

In line with this evidence, the stimulation by β-caryophyllene of G2/M checkpoint in response to the increased γ-H2AX by doxorubicin can be considered as a cytoprotective mechanism aimed at blocking cell cycle and repairing DNA-damage. Doxorubicin-induced DNA damage resulted strongly inhibited in normal cholangiocytes, despite the high ROS levels and GSH depletion. Conversely, in cholangiocarcinoma cells a lower genoprotection, which could be correlated to the lowered ROS levels, occurred. This suggests that the genoprotective effects of β-caryophyllene could be partly due to a direct potentiation of DNA repair systems, particularly in normal cholangiocytes. Indeed, cancer cells are known to carry defective DNA repair systems and a loss in mismatch repair function has been associated to doxorubicin resistance in cholangiocarcinoma cells [93,94]. This hypothesis could explain the different ability of the natural sesquiterpene to counteract the doxorubicin DNA damage in cancer and noncancerous cholangiocytes. However, the involvement of other direct or indirect genoprotective mechanisms cannot be excluded. Our results agree with previous evidence about the genoprotective properties of β-caryophyllene towards the DNA-damage induced by different environmental carcinogens [31,32] and pollutants [42,43]. The evidence that β-caryophyllene can act through both desmutagenic and bioantimutagenic mechanisms [95] also supports present results in cholangiocytes.

Under our experimental conditions, β-caryophyllene also activated proapoptotic signaling in cancer cells in response to the unrepairable DNA damage of doxorubicin (Figure 14A); conversely, in noncancerous cholangiocytes it activated survival signaling, likely as a consequence of a DNA-repair, which can inhibit apoptosis rate (Figure 14B).

Apoptosis is finely regulated by a permissive apoptotic environment, characterized by an altered redox homeostasis, involving a GSH depletion [96]. In numerous malignancies, apoptosis has been found downregulated by STAT3, a latent cytosolic transcription factor, activated by phosphorylation at tyrosine 705 (Tyr705) or serine 727 (Ser727) in response to different endogenous and exogenous stimuli and aberrantly phosphorylated in cancer cells, likely to facilitate their transformation [97]. Despite its sustaining role in cancer progression, drug resistance and metastasization, STAT3 can also act as a protective factor in normal cells, wherein it controls different genes, such as those involved in proliferation, survival and self-renewal [97]. STAT3 phosphorylation has been also associated to increased levels of ROS and γ-H2AX in damaged tissues, thus suggesting that this pathway underpins the DNA repair abilities [98]. Conversely, elevated STAT3 levels in cancer contribute to chemoresistance, thus suggesting that its inhibition can represent an interesting strategy for increasing cancer chemotherapy sensitivity [54].

Accordingly, under our experimental conditions, we found that phosphorylation of STAT3 at tyrosine 705 was promoted by doxorubicin in both cancer and noncancerous cells, being markedly overexpressed in Mz-ChA-1 cells (about a 10-fold higher expression with respect H69 cholangiocytes), whereas the combination with β-caryophyllene hindered its activation especially in H69 cells, achieving the normal expression in the control (Figure 14A,B). The ability of β-caryophyllene to affect the activation of phospho(Tyr705)-STAT3 agrees with our previous data in HepG2 cells [42], although the true inhibitory mechanisms (e.g., direct binding site antagonism or indirect interference) require to be further clarified.

On the basis of our results, we hypothesize that the natural sesquiterpene β-caryophyllene suppresses STAT3 phosphorylation, likely as a consequence of its genoprotective effects, thus blocking the activation of the survival signaling in cholangiocarcinoma cells and proapoptotic fate in normal cholangiocytes. Accordingly, Lee et al. [58] highlighted that anticancer drugs, like doxorubicin, can activate STAT3 to support cell survival and drug resistance. This reinforces the hypothesis that administering an anticancer drug in combination with a STAT3 inhibitor can enhance chemotherapy efficacy by disrupting the activation of this resistance mechanism.

Since STAT3 represents a key regulator of cancer survival and progression, several efforts have been made over the years in order to identify suitable direct or indirect inhibitors for anticancer therapies, although their application has been limited due to the low specificity for cancer tissues and the severe side effects [55]. Therefore, searching for new agents, including natural compounds or suitable delivery strategies to increase the cancer specificity and avoid undesirable effects, remains a great challenge. Several natural compounds showed the ability to inhibit STAT3 activation, leading to downregulation of survival genes, cell cycle arrest and apoptosis and also potentiating the cytotoxic effects of anticancer drugs [22,23,99,100,101,102,103,104,105]. Among them, curcumin exhibited anti-cholangiocarcinoma effects by the suppression of different transcription factor cascades, among which STAT3, NF-kB and AP-1 [106]. Similarly, the suppression of STAT3 signaling by honokiol inhibited liver cancer cell proliferation and potentiated the apoptotic effects of paclitaxel and doxorubicin [107].

Our data highlighted for the first time that an indirect inhibition of phospho(Tyr705)-STAT3 by the lowering of γ-H2AX, likely as a consequence of the activation of a DNA repair response, can be involved in the chemosensitizing and chemopreventive effects of β-caryophyllene towards doxorubicin in cancer and normal cholangiocytes. Interestingly, the inhibition of STAT3 by β-caryophyllene produced nontoxic effects in normal cholangiocytes, thus suggesting the involvement of specific mechanisms for cancer cells.

The here highlighted dual-acting role of β-caryophyllene, as both a chemosensitizer and a chemopreventive agent, also agrees with previous evidence. Particularly, Pavithra et al. [108] found that the natural sesquiterpene synergistically interacted with aromadendrene oxide 2 and phytol in skin epidermoid cancer cells, thus leading to apoptotic cell death, through ROS accumulation, caspase activation and PARP cleavage. Similar effects were registered in colon cancer cells [109]. Conversely, in normal neurons β-caryophyllene produced protective effects towards the damage of MPP+ (1-methyl-4-phenylpyridinium), by suppressing ROS generation and apoptosis signaling [39]. Also, β-caryophyllene was found able to counteract the doxorubicin-induced acute cardiotoxicity in rats through the inhibition of oxidative stress, DNA damage, apoptosis and inflammatory tissue response [39,110].

Altogether, this evidence strengthens our hypothesis about the ability of β-caryophyllene to both potentiate the antiproliferative activity of doxorubicin in cholangiocarcinoma cells and to prevent its toxicity in normal cholangiocytes. STAT3 seems to act as a final effector of an intricated network of apoptotic and survival stimuli, although further studies are required to deeply depict this intricated network.

## 5. Conclusions

Doxorubicin-based chemotherapy is known to possess powerful properties although it is limited by serious side effects and discomfort for administration route. Several strategies have been approached to overcome drawbacks of doxorubicin chemotherapy, among which alternative administration schedules and combination with adjuvant agents.

In the present study, we highlight the ability of a combination of doxorubicin with the natural chemosensitizer β-caryophyllene to suppress the proliferation of cholangiocarcinoma cells in both standard long-term and metronomic schedules and to prevent drug toxicity in normal cholangiocytes. A complex and intricated network seems to mediate these effects, thus leading to a final doxorubicin-chemosensitization in cholangiocarcinoma cells and chemoprevention in noncancerous cholangiocytes. A regulation of STAT3 signaling seems to represent the final effector of these networking effects, wherein the oxidative stress defenses, DNA damage response and cell cycle checkpoints finely cooperate.

This evidence strengthens our consolidated interest for the natural sesquiterpene β-caryophyllene as a dual-acting agent, i.e., chemosensitizer for chemotherapeutic drug and chemopreventive agent towards the toxic damage of a number of xenobiotics, including both pollutants and drugs, and suggests that a further characterization of its possible usefulness in chemotherapy can represent an important challenge for cancer research.

## Figures and Tables

**Table 1:** IC_50_ values of β-caryophyllene in cholangiocarcinoma Mz-ChA-1 cells and noncancerous H69 cholangiocytes under both long-term and metronomic schedules. In the last protocol, the cells were subjected to a short and/or double repeated exposure of 2 h to the test substance followed by a recovery time of 72 h. Data represent the mean ± SE (standard error) of at least two experiments in which each treatment was tested in triplicate (*n* = 6).

Time Exposure	β-Caryophyllene IC_50_ [µM] (CL)	
	Mz-ChA-1	H69
24 h	124.0 (105.0–176.2) *	147.6 (124.5–175.7)
72 h	90.0 (73.1–111.5) ^§^*	146.0 (98.7–211.5)
2 h	171.5 (80.5–213.5)	176.6 (153.7–202.9)
2 h double	139.5 (90.0–173.0) *	161.2 (131.4–197.6)

CL, confidential limits. ^§^ *p* < 0.01 (ANOVA + Multiple Dunnett’s comparison post-test), significantly lower than the IC_50_ value obtained after 24 h exposure. * *p* < 0.05 (ANOVA + Multiple Dunnett’s comparison post-test), significantly lower than the IC_50_ value after the single short treatment of 2 h.

**Table 2:** IC_50_ values of β-caryophyllene under both long-term and metronomic schedules in Mz-ChA-1 and H69 cells. In the last protocol, the cells were subjected to a short and/or repeated exposure of 2 h to the test substances followed by a recovery time of 72 h. Data represent the mean ± SE (standard error) of at least two experiments in which each treatment was tested in triplicate (n = 6).

Time Exposure	IC_50_ [µM] (CL ^a^)RR ^b^			
	Mz-ChA-1		H69	
	Doxorubicin	Doxorubicin + β-Caryophyllene	Doxorubicin	Doxorubicin + β-Caryophyllene
24 h	27.8 (18.0–42.4)	11.2 (7.4–16.6) °°	13.6 (5.8–31.2)	25.5 (12.0–45.0)
		2.5		0.5
72 h	3.8 (2.2–6.4) ^§^**	3.2 (1.2–8.6) ^§^**	4.0 (3.4–4.6) ^§^	3.6 (3.2–4.0)
		1.2		1.1
2 h	19.8 (8.2–45.4) ^§^	8.8 (2.6–18.2) ^§^°°	81.8 (69.8–126.2)	122.6 (50.0–301.0)
		2.3		0.7
2 h - double	11.6 (4.0–33.2) ^§^ *	5.8 (0.4–25.0) ^§**^°°	54.4 (39.4–75.0)	59.2 (46.2–76.0)
		2		0.9

^a^ CL, Confidential limits. ^b^ RR or reversal ratio: ratio between IC_50_ values of doxorubicin and its combination with β-caryophyllene. ^§^ *p* < 0.01 (ANOVA + Multiple Dunnett’s comparison post-test), significantly lower than the IC_50_ value after 24 h. *p < 0.05 and **p < 0.01 (ANOVA + Multiple Dunnett’s comparison post-test), significantly lower than the IC_50_ value after a single short treatment of 2 h. ^°°^*p* < 0.01 (ANOVA + Multiple Dunnett’s comparison post-test), significantly lower than the IC_50_ value of doxorubicin in the same schedule.

## Supplementary Materials

The following are available online at https://www.mdpi.com/2073-4409/9/4/858/s1, Figure S1: Schedule of the long-term exposures of 24 and 72 h. Figure S2: Schedule of the metronomic treatment. Figure S3: Effect of a single administration of β-caryophyllene (50 and 100 mg/kg b.wt./i.p. in olive oil; single dose), doxorubicin (3 mg/kg b.wt./i.p. in physiological solution; single dose) and their combinations on body, heart and liver weight in Sprague Dawley rats. Figure S4: Cytotoxic effects of doxorubicin (0.2–100 μM) and its combination with β-caryophyllene (50 μM) in human prostatic carcinoma PC3 cells, lacking in the expression of STAT3, after a single long-term exposure of 24 h.
